# Analogues of ERβ ligand chloroindazole exert immunomodulatory and remyelinating effects in a mouse model of multiple sclerosis

**DOI:** 10.1038/s41598-018-37420-x

**Published:** 2019-01-24

**Authors:** Hawra Karim, Sung Hoon Kim, Kelli Lauderdale, Andrew S. Lapato, Kelley Atkinson, Norio Yasui, Hana Yamate-Morgan, Maria Sekyi, John A. Katzenellenbogen, Seema K. Tiwari-Woodruff

**Affiliations:** 1 Division of Biomedical Sciences, UCR School of Medicine, Riverside, CA 92521 USA; 2 Department of Chemistry, University of Illinois at Urbana-Champaign, Urbana, IL 61801 USA; 3 Center for Glia Neuronal Interaction, UCR School of Medicine, Riverside, CA 92521 USA

## Abstract

Pharmaceutical agents currently approved for the treatment of multiple sclerosis reduce relapse rates, but do not reverse or prevent neurodegeneration nor initiate myelin repair. The highly selective estrogen receptor (ER) β ligand chloroindazole (IndCl) shows particular promise promoting both remyelination while reducing inflammatory cytokines in the central nervous system of mice with experimental autoimmune encephalomyelitis. To optimize these benefits, we developed and screened seven novel IndCl analogues for their efficacy in promoting primary oligodendrocyte (OL) progenitor cell survival, proliferation, and differentiation *in vitro* by immunohistochemistry. Two analogues, IndCl-*o*-chloro and IndCl-*o*-methyl, induced proliferation and differentiation equivalent to IndCl and were selected for subsequent *in vivo* evaluation for their impact on clinical disease course, white matter pathology, and inflammation. Both compounds ameliorated disease severity, increased mature OLs, and improved overall myelination in the corpus callosum and white matter tracts of the spinal cord. These effects were accompanied by reduced production of the OL toxic molecules interferon-γ and chemokine (C-X-C motif) ligand, CXCL10 by splenocytes with no discernable effect on central nervous system-infiltrating leukocyte numbers, while IndCl-*o*-methyl also reduced peripheral interleukin (IL)−17. In addition, expression of the chemokine CXCL1, which is associated with developmental oligodendrogenesis, was upregulated by IndCl and both analogues. Furthermore, callosal compound action potential recordings from analogue-treated mice demonstrated a larger N1 component amplitude compared to vehicle, suggesting more functionally myelinated fibers. Thus, the *o*-Methyl and *o*-Chloro IndCl analogues represent a class of ERβ ligands that offer significant remyelination and neuroprotection as well as modulation of the immune system; hence, they appear appropriate to consider further for therapeutic development in multiple sclerosis and other demyelinating diseases.

## Introduction

Multiple sclerosis (MS) is an autoimmune, demyelinating, and neurodegenerative disease of the central nervous system (CNS) with no known cause or cure. Experimental autoimmune encephalomyelitis (EAE) recapitulates the inflammation, demyelination, and neurodegeneration observed in MS and is among the most common inducible animal models of MS^1^. The EAE model has been used to develop many of the currently approved MS treatments, including interferon (IFN)-β, glatiramer acetate, fingolimod, and the anti- cluster of differentiation (CD) 20 monoclonal antibody, ocrelizumab^1,2^. However, although these therapeutics attenuate inflammation, they neither prevent neurodegeneration nor initiate remyelination.

Accumulating evidence indicates that estrogens are both neuroprotective and immunomodulatory, making them attractive candidates for the treatment of MS. Estrogens skew the inflammatory T helper (Th) 1 response prevalent in MS towards an anti-inflammatory Th2 profile^3,4^. Furthermore, in preclinical studies, treatment with pregnancy levels of the placenta-derived estrogenic hormone estriol attenuated EAE disease severity^5,6^. However, although they display immense potential for treating MS, endogenous estrogen therapy possesses several undesirable or deleterious side effects^7^. In addition to feminizing male recipients, treatment with endogenous estrogens increase the risk of developing breast and endometrial cancers in females^7^. Importantly, the carcinogenic effects of estrogens are mediated through estrogen receptor (ER)α and not ERβ, suggesting that therapies targeting specific ER subtypes may impart the benefit of estrogen treatment, while circumventing these side effects^8^.

In support of this notion, chloroindazole (IndCl), a halogen-substituted phenyl-2H-indazole core with up to 100-fold relative binding affinity for ERβ over ERα^9^, has demonstrated promise as an immunomodulatory, pro-myelinating, and neuroprotective agent in mouse models of MS^10,11^. In C57BL/6 mice with EAE, IndCl attenuated disability scores and improved rotarod performance^10,12^. This was accompanied by reduced frequency of CNS-infiltrating CD45^+^ leukocytes and decreased production of inflammatory cytokines by antigen reactivated splenocytes^10,11^. Similarly, IndCl suppressed lipopolysaccharide or interleukin (IL)−1β-induced upregulation of inducible nitric oxide synthase, IL-1β, IL-6, and IL-23, in cultured human and murine microglia and astrocytes^12,13^.

In addition to reducing inflammation, IndCl and other ERβ ligands act on oligodendrocytes (OLs) directly to support their proliferation, differentiation, and overall myelination activity^12,14^. Mice with EAE that received IndCl treatment showed increased myelin basic protein (MBP) and mature OL numbers in the spinal cord and corpus callosum (CC)^10,11^. Concomitantly, IndCl increased the number of actively dividing OL progenitor cells (OPCs) in the subventricular zone neurogenic niche and adjacent white matter lesions^10^. Critically, electrophysiological evaluation showed that the pro-myelinating effects of IndCl correlated with functional recovery, as compound action potential recordings from treated mice exhibited improved callosal axon conduction^10^. Additionally, unlike estrogens or ERα ligands, IndCl may directly oppose oncogenesis and is anti-proliferative in several disease models^15^. For instance, in a recent study, IndCl reduced inflammation and inhibited the establishment of endometrial lesions in a mouse model endometriosis^16^.

**Figure 1:**
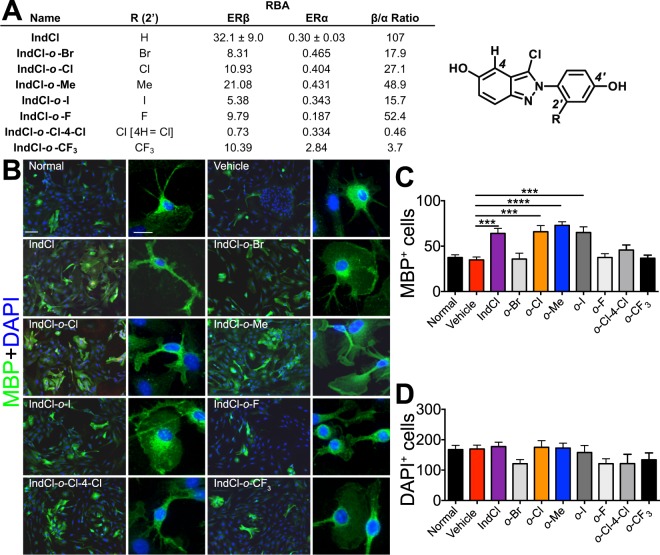
Estrogen receptor β (ERβ) ligand IndCl and analogue structure and effect on cell survival. **(A)** Estrogen receptor binding affinity for IndCl and seven analogues, IndCl-*o*-Br, IndCl-*o*-Cl, IndCl-*o*-Cl, IndCl-*o*-IMe IndCl-*o*-I, IndCl-*o*-F, IndCl-*o*-Cl-4-Cl and IndCl-*o*-CF_3_ (Fig. 1A), which were synthetized by techniques shown in Supplementary Fig. S1. **(B)** Some estrogen receptor β (ERβ) ligands increase primary mouse oligodendrocyte differentiation: Representative images of primary OPCs/OLs from wells containing differentiating media alone (normal media), vehicle, positive control IndCl, or the 7 different IndCl analogues. OLs were immunostained with myelin basic protein (MBP; green) and co-stained with nuclear stain DAPI (blue). **(C-D)** Effects of treatment on the number of MBP^+^ OLs and the number of total cells were quantified. Analogue IndCl-*o-*Cl, IndCl-*o*-Me and IndCl-*o-*I showed a significant increase in the number of MBP^+^ OLs with an increase in the percentage of branched OLs, compared to vehicle treated cells. No significant differences in total number of cells were observed between groups. There were 3 wells/treatment group. n = 3 independent experiments were performed. One-Way ANOVA with Dunnett’s multiple comparisons test.

In this report, we have investigated the therapeutic efficacy of IndCl analogues using cell culture, mouse behavior, functional electrophysiology, and CNS histology. In doing so, we have included for comparison some IndCl analogues in the hopes of discovering a remyelinating ERβ ligand that would be suitable for pre-clinical development and transition from bench to bedside. Our interest in including IndCl analogues is supported by the rather different activity found between similar selective ERβ ligands such as diarylpropionitrile and IndCl^10^. To this end, we prepared and evaluated seven IndCl analogues that were modified to contain an additional substituent on the 2′ position of the 4′-hydroxyphenyl ring, and one having an additional chlorine substituent on the 4 position **(**Fig. 1**)**. These seven analogues, all of which exhibited ERβ-preferential binding affinities, were initially screened in primary OPC cultures for survival, proliferation and differentiation. From this initial set, only two, IndCl-*o*-chloro (IndCl-*o*-Cl) and IndCl-*o*-methyl (IndCl-*o*-Me), showed activity comparable or superior to IndCl and were thus selected for *in vivo* testing in mice with EAE. Herein, we delineate their immunomodulatory and neuroprotective effects.

## Materials and Methods

All methods were carried out in accordance with relevant guidelines and regulations. All experimental protocols were approved, and animals were maintained as mandated by the UC-Riverside Office of Research Integrity and the Institutional Animal Care and Use Committee.

### Synthesis of IndCl analogues and analogues

IndCl and six analogues, IndCl-*o*-Br, IndCl-*o*-Cl, IndCl-*o*-Me, IndCl-*o*-I, IndCl-*o*-F, IndCl-*o*-Cl-4-Cl and IndCl-*o*-CF_3_ (Fig. 1A), were synthesized by routes shown in Supporting Information Fig. S1. Experimental protocols and evidence for product identity and purity are also given in the Supporting Information.

### Primary OPC Cultures

Primary OL progenitor cells (OPCs), isolated from postnatal day (p) 1 C57BL/6 male and female mouse cortices as described previously, were treated with 10 nM ligands in differentiating medium for 3 days.

### Primary astrocyte cultures and OPC treatment

Astrocyte cultures were prepared from the cerebral cortex of p0-p4 C57BL/6 pups^11,17^. Purified astrocyte cultures were then treated with 13 ng/ml IL-1β, 10 nM vehicle, IL-1β + IndCl-*o*-Cl, IL-1β + IndCl-*o*-Me or media for 48 hours. Astrocyte conditioned media (ACM) from the various conditions was used for enzyme-linked immunosorbent assay (ELISA) and primary OL culture treatment with and without 100 nM CXCR2 antagonist, SB225002 (Tocris, Minneapolis, MN) for 48 hours. Cells were then fixed with 10% formalin and analyzed.

### Enzyme-linked Immunosorbent Assay (ELISA)

CXCL1 concentrations (pg/ml) in astrocyte culture supernatant were measured using an ELISA murine CXCL1 kit (PeproTech US, Rocky Hill, NJ) according to the manufacturer’s instructions. The absorbance was read in a microplate reader (Bio-Rad Laboratories, Hercules, CA) set to 405 nm with 605 nm wavelength corrections.

### EAE induction

Active EAE was induced in eight-week-old female C57BL/6 and Thy1-YFP mice as previously described (one of three representative EAE experiments).

### Treatment

Drugs (IndCl, IndCl-*o*-Cl, and IndCl-*o*-Me) were dissolved in vehicle solution (10% ethanol and 90% Miglyol 812N (Cremer; Sasol, Germany) at a dose of 5 mg/kg/day animal weight or vehicle. Treatment (100 μL/day by subcutaneous injection) was initiated at post-immunization Day8 or Day17. As a positive control, mice were given 0.05 mg/kg/day estradiol (E2) beginning at Day0 (1^st^ MOG injection). n = 10 sex and age matched animals for normal, preE2, postE2, IndCl, IndCl-*o*-Cl, and IndCl-*o*-Me groups (60 mice in total).

### Rotarod behavioral assay

Motor behavior was quantified up to twice per week for each mouse using a rotarod apparatus (Med Associates, Inc., St. Albans, VT) and was performed (only on the early treatment EAE groups) as previously described^10^.

### Histological Preparation of Tissues

Mice were deeply anesthetized by isoflurane (Piramal Healthcare) inhalation and perfused transcardially with phosphate buffered saline (PBS), followed by 10% formalin for (ThermoFisher Scientific) to fix tissues. 10% formalin post fixed, 30% sucrose cryoprotected brains and spinal cords were cut into serial 40-μm coronal sections and subjected to immunohistochemistry (IHC), following a previously described protocol^10,18^.

### Splenocyte Isolation and Cytokine Analysis

Spleens were dissected from anesthetized mice prior to intracardiac perfusion and mechanically dissociated into a single cell suspension in cold RPMI 1640 supplemented with pyruvate, L-glutamine, and 10% fetal bovine serum (henceforth referred to as RPMI). Red blood cells were lysed by incubation with ACK buffer (VWR), washed, counted, and suspended in RPMI for cytokine analysis. Splenocytes were then stimulated with 25 µg/ml MOG_35–55_ and supernatants were collected 48 hours later^11,14,19^. Levels of the following cytokines and chemokines present in culture supernatants were evaluated using a 20 plex Cytokine Mouse Magnetic Panel for Luminex (ThermoFisher Scientific) run on a Luminex MAGPIX detection system; pro-inflammatory cytokines: GM-CSF, IFNγ, IL-1α, IL-1β, IL-2, IL-6, IL-12, IL-17, Tumor necrosis factor (TNF)α, and Vascular endothelial growth factor (VEGF); anti-inflammatory cytokines: IL-4, IL-5, IL-10, IL-13, and fibroblast growth factors (FGF); chemokines: CXCL10, CXCL1, CCL2, CXCL9, and CCL3 were determined (Thermo Fisher Scientific).

### Transmission electron microscopy

Mice were perfused with PBS as above followed by paraformaldehyde/glutaraldehyde to preserve ultrastructure and Epon embedded as previously described^20^. Serial ultrathin sections of Epon-embedded CC were stained with uranyl acetate-lead citrate were used for electron microscopy analysis^21^. G-ratio was measured using Fiji v1.0 Software (NIH).

### Confocal microscopy

Immunostained brain sections containing CC and thoracic spinal cord sections Z-stack projected images were acquired using an Olympus BX61 spinning disk confocal microscope (Olympus America Inc.) and SlideBook 6 (Intelligent Imaging Innovations, Inc.) or CellSens software (Olympus America Inc.). Images were quantified using unbiased stereology as previously described using Fiji v1.0 Software (NIH).

### Electrophysiology

To assess functional conductivity across the CC, electrophysiological recordings of compound action potentials (CAPs) were measured as previously described^20,22^. Electrophysiology data were analyzed using Clampfit 10.4 software (Molecular Devices, Sunnyvale, CA) and OriginPro 2016 64Bit (OriginLab Corporation).

### Statistical analysis

Statistical significance was determined for all data sets using GraphPad Prism 6 (GraphPad Software) as previously described^10,11,23^. All data are presented as mean ± SEM for 2–3 independent experiments. Differences were considered significant at *P < 0.05, **P < 0.01, ***P < 0.001, ****P < 0.0001.

## Results

### Treatment with IndCl analogues stimulates differentiation of OPCs *in vitro*

Primary OPC cultures were used as a cell-based assay to characterize the differentiating effects of the new IndCl analogues and to select those most suitable for more extensive studies to be performed in comparison with the parent ligand, IndCl. Primary OPCs prepared from mouse neonatal cortex were treated with one of seven IndCl analogues or control at a concentration of 10 nM^24,25^. After 72 hours, cells were fixed and labeled with an antibody against MBP and the number of MBP^+^ labeled OLs were counted. (Fig. 1B,C)^9,12^. Treatment with IndCl, IndCl-*o*-Cl, IndCl-*o*-Me, and IndCl-*o*-I increased the number of MBP^+^ cells and branching processes, indicative of efficient OL differentiation as compared to vehicle-treated cultures (Fig. 1B,C). By contrast, the other 4 analogues did not affect cell differentiation as compared to vehicle-treated groups (Fig. 1B,C). The total number of cells in culture was not altered by any treatment (Fig. 1B,D)

### IndCl analogues ameliorate EAE severity more effectively than IndCl and improve rotarod performance without affecting uterine weight

**Figure 2:**
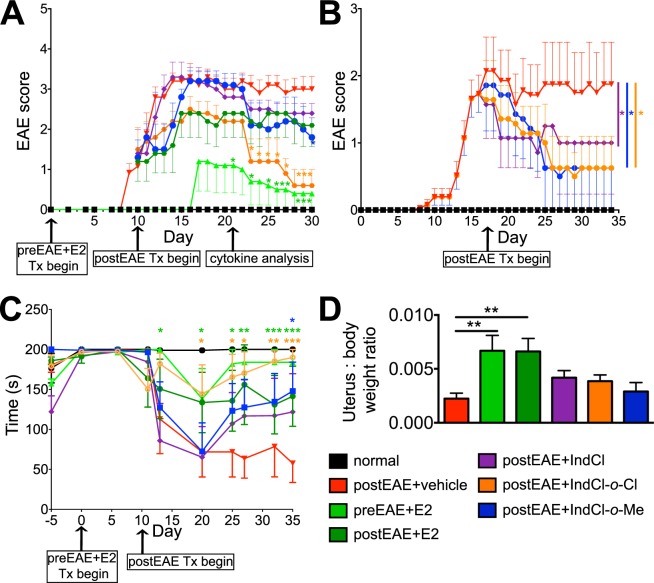
Therapeutic treatment with IndCl analogues ameliorates EAE disease, improves rotarod performance, and does not increase uterine weight. **(A**,**B)** Mice were immunized with MOG_35–55_. Normal mice did not receive MOG_35–55_ or treatment. **(A)** Therapeutic treatment with ERβ ligands, IndCl (5 mg/kg/d; purple), IndCl-*o*-Cl (5 mg/kg/d; orange) and IndCl-*o*-Me (5 mg/kg/d; blue) and 17β-estradiol (E2; 0.05 mg/kg/d; dark green) began at the onset of clinical disease (day 8) until day 30. Prophylactic E2 (0.05 mg/kg/d; light green) delayed onset of clinical disease. Vehicle-treated EAE mice (red) displayed onset of clinical disease symptoms between days 7–10, with disease severity peaking around day 15. During peak disease, IndCl (purple), IndCl-*o*-Cl (orange) and IndCl-*o*-Me (blue) treatment did not significantly affect EAE clinical symptoms, but decreased disease progression over time. **(B)** Therapeutic treatment with ERβ ligands, began at peak disease (day 17) and was continued daily till day 35. Vehicle-treated EAE mice displayed onset of clinical disease symptoms around day 9–10 with peak disease occurring on day 17. All ERβ ligands significantly attenuated clinical disease severity compared to vehicle treatment. One of two representative EAE experiments is shown. n = 8–10 mice/group, Two-Way ANOVA with Dunnett’s multiple comparisons test. **(C)** To assess motor function, mice were subjected to the rotarod motor performance test. Vehicle-treated EAE mice displayed an abrupt and consistent decrease in time (seconds) remaining on the rotarod. While EAE mice treated with IndCl-*o*-Cl remained on the rotarod significantly longer indicative of improved motor function. Data are representative of experiments repeated three times. n = 8–10 mice/group, Ordinary One-Way ANOVA with Dunnett’s multiple comparisons test. **(D)** Assessment of post-perfusion uterus to body weight ratios from normal and EAE mice treated with prophylactic E2 (dark green), or therapeutic E2 (light green), IndCl (purple), IndCl-*o*-Cl (orange) and IndCl-*o*-Me (blue). Both prophylactic and therapeutic E2 treated female mice showed a fourfold increase of uterus to body weight ratio with no differences between all other treatment groups. n = 8–10 mice/group, One-Way ANOVA with Dunnett’s multiple comparisons test analysis.

IndCl has been shown to reduce motor disability in EAE mice when administered prophylactically or therapeutically^10^. Having established that IndCl-*o*-Cl and IndCl-*o*-Me exhibited comparable effects to IndCl *in vitro*, their impact was next evaluated *in vivo* using eight-week-old female C57BL/6 mice in which EAE had been induced following an established protocol^26^. As a positive control for non-ER isoform specific estrogenic signaling, mice were given prophylactic E2 subcutaneously at the time of initial immunization with MOG_35–55_ peptide, which continued throughout the course of experiments (PreEAE + E2 group). All other groups received therapeutic daily subcutaneous doses of vehicle (PostEAE + vehicle), E2 (PostEAE + E2), IndCl (PostEAE + IndCl), IndCl-*o*-Cl (PostEAE + IndCl-*o*-Cl), or IndCl-*o*-Me (PostEAE + IndCl-*o*-Me) that began at the onset of clinical symptoms (day 8; Fig. 2A) or at peak disease (day 17; Fig. 2B) and continued throughout the course of experiments. The timing of the different dosage regimens is illustrated schematically in Fig. 2A,B.

Disease course was greatly attenuated in mice that received prophylactic but not therapeutic E2 treatment, compared to those that received vehicle only, in which accumulating motor deficits appeared between post-immunization days 8–12 and persisted for the duration of experiments (Fig. 2A). Both therapeutic IndCl-*o*-Cl and IndCl-*o*-Me, when administered at onset of clinical symptoms significantly reduced EAE clinical scores beginning at post-immunization day 23, roughly two weeks after treatment (Fig. 2A). This is consistent with previously published reports using IndCl and other ERβ ligands, which demonstrated significant protective effects at later stages of disease^10,27–29^. IndCl and analogue treatment administered at peak disease (day 17), also reduced EAE clinical severity significantly compared to vehicle treatment (Fig. 2B).

As a complementary assay of motor function, mice (from Fig. 2A set) were tested on a rotarod device following a previously described protocol^10^. Normal mice and those that received prophylactic E2 did not fall off the rotarod within the time allotted, whereas vehicle and therapeutic E2 and IndCl treated mice had a tendency to fall from the cylinder abruptly. Both IndCl-*o*-Cl and IndCl-*o*-Me treatment improved rotarod performance compared to vehicle and IndCl by day 20 post-immunization with IndCl-*o*-Me treatment group exhibiting the greatest improvement in motor function (Fig. 2C).

Estrogens increase uterine weight by acting primarily through ERα^30^. In order to determine whether analogues tested possessed ERα signaling properties that could contribute to the improved motor performance observed, uterine weight was assessed. As expected, prophylactic and therapeutic E2 treatment significantly increased uterus to bodyweight ratios (Fig. 2D). In contrast, neither IndCl nor its analogues significantly increased this ratio (Fig. 2D).

### IndCl analogues increase myelination in spinal cord white matter during EAE

**Figure 3:**
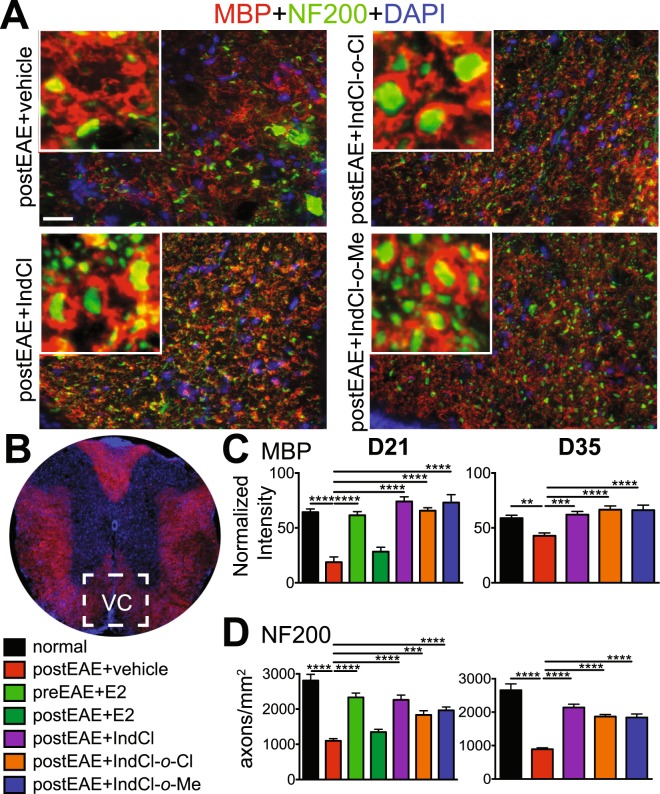
Therapeutic treatment with IndCl analogues improves myelination in the spinal cord of peak disease EAE animals. **(A)** Representative 40x magnification coronal images of the ventral column of thoracic spinal cord (area delineated by white square in **3B**), showing axons stained with MBP (red), neurofilament 200 (NF200; green) and nuclear DAPI stain (blue). Inset (white box) depicts zoomed in magnification images to show myelin wrapped axons. Scale bar represents 10 µM. **(C,D)** Quantification of MBP intensity and NF200 axons was performed at day 21 (Fig. 2A) and day 35 (Fig. 2B) postEAE. **(C)** Vehicle-treated EAE mice exhibited significantly decreased MBP intensity which was maintained at near normal levels with prophylactic E2 at day 21 and therapeutic IndCl, IndCl-*o*-Cl and IndCl-*o*-Me treatment at both time points. **(D)** Quantification of NF200 numbers reveals a significant decrease in the number of axons in the vehicle-treated mice. Prophylactic E2 treatment showed significant recovery of axons at day 21 with therapeutic IndCl, IndCl-*o*-Cl and IndCl-*o*-Me treatment exhibiting significantly increased NF200+ axon staining at both time points. n = 5–7 mice/group, One-Way ANOVA with Dunnett’s Multiple Comparisons Analysis.

Treatment with IndCl or other ERβ ligands enhances axon myelination within CNS white tracts of mice with EAE^10,14,27^. To establish the pro-myelinating effects of the IndCl analogues tested, thoracic ventral column white matter (Fig. 3B) was assessed for MBP immunoreactivity from mice treated during onset of clinical symptoms (day 8) or peak disease (day 17). Mice that received vehicle treatment showed significantly reduced MBP staining intensity as well as loss of NF200 + axons compared to normal, consistent with previous studies^10,18,26^ (Fig. 3A–D, SI appendix Fig. S1). Therapeutic IndCl, IndCl-*o*-Cl, and IndCl-*o*-Me treatment, either on the onset of disease or during peak disease, and prophylactic E2 treatment, increased MBP staining intensity and NF200^+^ axon numbers relative to vehicle. All IndCl ligands tested had comparable remyelinating effects when treatment was started early disease or peak disease (Fig. 3A–D, SI appendix Fig. S1).

### IndCl analogues modify peripheral cytokine and chemokine responses in EAE

During MS and EAE, peripherally activated leukocytes secrete inflammatory cytokines and chemokines as they migrate into the CNS, where they contribute to demyelination and axon damage^31^. To characterize the effects of IndCl analogues on the peripheral immune response, splenocytes were isolated from mice 21 days post-immunization (from Fig. 2A set) and stimulated *ex vivo* with MOG_35–55_ peptide for cytokine and chemokine analysis using a magnetic bead-based 20-plex cytokine/chemokine detection assay. Effects on cytokines related to inflammation, CD4^+^ T cell polarization, immune regulation, and chemokines associated with OL apoptosis and myelination that were measured in collected supernatants are presented below.

#### Pro-inflammatory Cytokines

**Figure 4:**
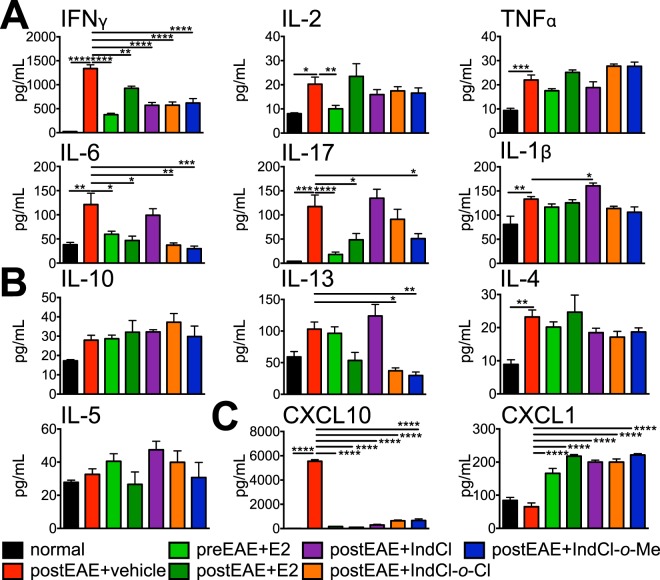
Therapeutic treatment with IndCl analogues decreases pro-inflammatory cytokine IFNγ, pro-inflammatory chemokine CXCL10 and increases chemokine CXCL1 production by peripheral immune cells during peak EAE disease. Cytokine production by MOG_35–55_-stimulated splenocytes was assessed from EAE mice culled on post induction day 21 (from Fig. 2A set). **(A)** Vehicle-treated mice exhibited significantly increased levels of pro-inflammatory cytokines: IFNγ, IL-2, TNFα, IL-6, IL-17, and IL-1β compared to normal controls. Prophylactic E2 significantly decreased IFNγ, IL-2, IL-6 and IL-17 levels compared to vehicle, with therapeutic E2 decreasing IFNγ, IL-6, IL-17 levels. Therapeutic IndCl treatment significantly decreased IFNγ levels compared to vehicle. Treatment with IndCl-*o*-Cl significantly decreased IFNγ and IL-6, with IndCl-*o*-Me significantly decreasing IFNγ, IL-17, IL-6 levels compared to vehicle. **(B)** Anti-inflammatory cytokine production of IL-10, IL-13, IL-4, and IL-5 revealed no significant differences in any of the treatment groups compared to vehicle, except for IL-13 which significantly decreased in IndCl-*o*-Cl and IndCl-*o*-Me treated mice. **(C)** Vehicle-treated mice exhibited significantly elevated levels of CXCL10 compared to normal controls. Prophylactic E2 and therapeutic treatment with E2 and ERβ ligands significantly reduced CXCL10 levels compared to vehicle. Prophylactic and therapeutic treatment with E2 and ERβ ligands showed a significant increase in chemokine CXCL1 levels compared to vehicle. Data are representative of experiments repeated twice. n = 4–6 mice/group, Kruskal Wallis Analysis with Dunn’s Multiple Comparisons Analysis and One-Way ANOVA with Dunnett’s Multiple Comparisons Analysis.

As expected, splenocytes from vehicle-treated mice exhibited greater production of IFN*γ*, IL-2, TNFα, IL-6, IL-17 and IL-1β relative to normal. Prophylactic E2 reduced IL-2, IL-6, IL-17, and IFN*γ* concentrations, but had no effect on IL-1β or TNFα, whereas therapeutic E2 reduced IL-6, IL-17, and IFN*γ* only. IndCl and both the *o*-Me and *o*-Cl analogues decreased IFN*γ* concentrations in supernatants relative to vehicle, while decreased IL-6 production was observed in splenocytes of all treatment groups except for IndCl. IndCl-*o*-Me stood out among ERβ ligands tested as also reducing IL-17 production. None of the ERβ ligands included in this study affected IL-2 or TNFα. Interestingly, IndCl treatment alone led to increased IL-1β production compared to vehicle (Fig. 4A).

#### Anti-inflammatory Cytokines

Skewing the adaptive immune response toward a Th2 profile, which is characterized by production of cytokines such as IL-4, IL-5, and IL-13, ameliorates EAE disability^32^. Therefore, concentrations of these cytokines, along with the key anti-inflammatory and immunoregulatory cytokine IL-10^33^, in supernatants were assessed. Splenocytes from vehicle-treated mice exhibited increased IL-4 production compared to normal, but IL-10, IL-13, and IL-5 levels remained unchanged. Neither prophylactic nor therapeutic E2 significantly altered Th2 cytokine or IL-10 production relative to vehicle. Similarly, IndCl had no effect on cytokine concentrations. In contrast to IndCl, splenocytes from IndCl-*o*-Cl and IndCl-*o*-Me-treated mice exhibited attenuated IL-13 production compared to vehicle (Fig. 4B).

#### Chemokines

CXCL1 and CXCL10 are leukocyte chemoattractants with critical, but largely divergent effects on OPC survival. CXCL1 signaling through its receptor, CXCR2, is essential for homeostatic white matter development, OPC proliferation, and survival^34^. In contrast, CXCL10 induces OPC cell death *in vitro*, which is augmented by the addition of IFN*γ*^34^. Splenocytes from vehicle-treated mice displayed no change in CXCL1, but significantly upregulated CXCL10 production compared to normal. Splenocytes from prophylactic and therapeutic E2 treated mice produced increased concentrations of CXCL1 and decreased CXCL10 relative to vehicle. Similarly, splenocytes from both IndCl and analogue-treated mice exhibited increased CXCL1 and decreased CXCL10 levels compared to vehicle-treated animals (Fig. 4C).

### IndCl analogue treatment does not affect leukocyte infiltration or astrogliosis in thoracic spinal cord white matter

IndCl has been shown to reduce several indicators of inflammation during EAE, including staining intensity of the pan-leukocyte marker CD45 and the degree of glial fibrillary acidic protein (GFAP)^+^ astrogliosis present in the dorsal column white matter^10^. To assess whether IndCl analogues exert similar anti-inflammatory effects, CD45^+^ leukocyte, and GFAP^+^ astrocytes were assessed in thoracic spinal cord dorsal column sections from normal and EAE mice sacrificed at day 21 (when treatment was started early EAE) and day 35 (when treatment was started peak EAE) postEAE.

#### Leukocytes

**Figure 5:**
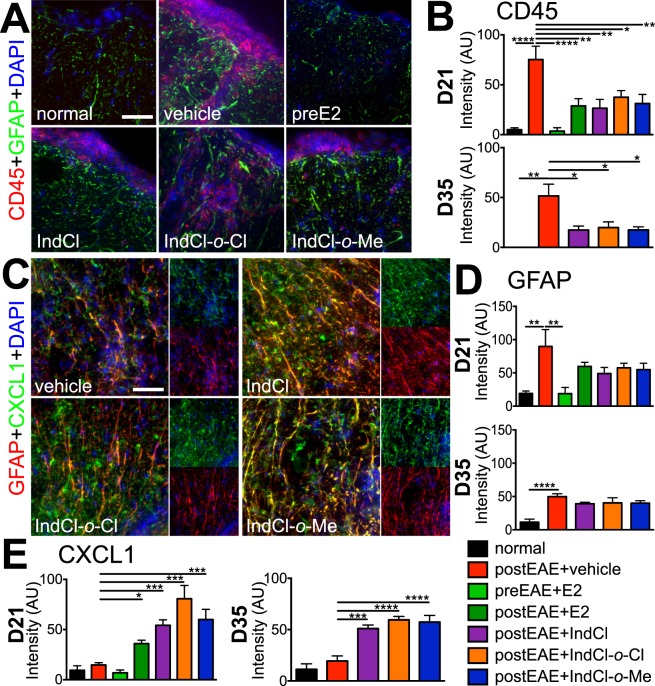
Therapeutic treatment with IndCl analogues does not decrease CNS inflammation, but increases CXCL1 production by astrocytes in the CNS. **(A)** Representative 40x magnification images of the spinal cord dorsal column reveals increased intensity of cluster of differentiation (CD)45 and glial fibrillary acidic protein (GFAP), in vehicle-treated EAE mice compared to normal control mice. **(B)** Prophylactic E2 (green) and therapeutic E2 (dark green) treatment decreased CD45 intensity at day 21 postEAE. IndCl (purple), IndCl-*o*-Cl (orange) and IndCl-*o*-Me (blue) significantly decreased CD45 intensity at both time points compared to vehicle-treated mice. **(C)** Representative 40x magnification coronal images of the ventral column of thoracic spinal cord collected at peak disease (day 21). Sections collected from vehicle, IndCl, IndCl-*o*-Cl and IndCl-*o*-Me were immunostained with chemokine (C-X-C motif) ligand 1 (CXCL1; green), glial fibrillary acidic protein (GFAP; red), and nuclear stain (DAPI; blue). Scale bar represents 10 µM for A&C. **(D)** Quantification of the relative fluorescence intensity of GFAP from normal, vehicle, prophylactic E2, therapeutic IndCl, IndCl-*o*-Cl and IndCl-*o*-Me treated EAE mice. Vehicle-treated mice exhibited increased GFAP fluorescence intensity at both time points that was significantly decreased only with prophylactic E2 treatment at day 21 ERβ ligand treatment with IndCl, IndCl-*o*-Cl and IndCl-*o*-Me exhibited similar degrees of intensity of GFAP as vehicle-treated EAE mice. **(E)** Quantification of the relative fluorescence intensity of CXCL1 revealed a significant increase in CXCL1 intensity in therapeutic E2 at day 21 and therapeutic IndCl, IndCl-*o*-Cl and IndCl-*o*-Me at both time points as compared to vehicle-treated EAE mice. n = 5–8 mice/group, One-Way ANOVA with Dunnett’s Multiple Comparisons Analysis.

In mice given vehicle only, dorsal column white matter displayed extensive CD45^+^ infiltration into spinal cord parenchyma, with staining intensity significantly elevated relative to normal controls. Prophylactic and therapeutic treatment with E2, as well as therapeutic treatment with IndCl and the analogues, either on the onset of disease or during peak disease, significantly decreased CD45^+^ staining intensity compared to vehicle-treated mice (Fig. 5A,B, SI appendix Fig. S2).

#### Astrogliosis

GFAP^+^ staining intensity was significantly increased in dorsal column white matter from vehicle-treated mice, indicating widespread astrogliosis. In line with its effects on other measures of inflammation, prophylactic E2 significantly reduced GFAP^+^ staining intensity. By contrast, therapeutic E2, IndCl, or IndCl analogue treatment, either on the onset of disease or during peak disease, did not modify GFAP^+^ staining intensity at either of the postEAE time points (Fig. 5C,D, SI appendix Fig. S2).

### Therapeutic IndCl analogues enhance astrocytic CXCL1 expression during EAE

Under inflammatory conditions, such as those generated by MS, astrocytes undergo NF-κB-dependent upregulation of CXCL1, which is thought to recruit OPCs to the site of demyelinating injury^35,36^. Thus, having observed that IndCl raised its production by peripheral leukocytes, we next evaluated CXCL1 expression in thoracic ventral column white matter. Vehicle-treated mice exhibited CXCL1^+^ staining intensity was comparable to normal (Fig. 5C,E; SI appendix Fig. S3). In contrast with its effect on CXCL1 production by peripheral leukocytes, prophylactic E2 treatment caused no change in CXCL1 staining intensity relative to vehicle (Fig. 5C,E, SI appendix Fig. S3). However, there was a small but significant increase in CXCL1 intensity with therapeutic E2 treatment. Notably, IndCl, IndCl-*o*-Cl, and IndCl-*o*-Me treatment, either on the onset of disease or during peak disease, significantly increased ventral column CXCL1 staining intensity compared to vehicle, with staining intensity appearing to co-localize more extensively with GFAP^+^ astrocytes (Fig. 5C,E, SI appendix Fig. S3).

### IndCl analogues upregulate astrocytic CXCL1 and stimulate OPC survival and differentiation *in vitro*

**Figure 6:**
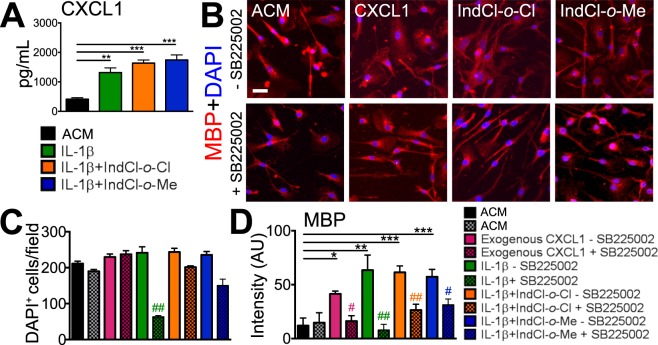
ERβ-induced astrocytic CXCL1 upregulation increases OL survival and differentiation. **(A)** Primary astrocytes were cultured with media alone, 13 ng/mL IL-1β, 10 nM IL-1β + IndCl-o-Cl, or 10 nM IL-1β + IndCl-o-Me for 48 hours, after which supernatant (astrocyte conditioned media; ACM) was collected and CXCL1 concentration (pg/ml) was measured by ELISA. Astrocytes treated with IL-1β, IL-1β + IndCl-o-Cl, or IL-1β + IndCl-o-Me significantly increased CXCL1 levels compared to untreated astrocytes. **(B)** Representative 20x images of primary OPCs cultured in the presence of ACM from (A) for 48 hours immunostained for MBP (red) and DAPI (blue) with and without 100 nM CXCR2 antagonist, SB225002. Scale bar represents 10 µM. **(C)** Quantification of DAPI + cells from (B) revealed a significant decrease in total cells with IL-1β treatment compared to ACM alone in the presence of SB225002 (# indicates significance between cultures treated with SB225002 vs those without). There were no significant differences between all other treatment groups. **(D)** Quantification of MBP intensity from (B) showed increased staining intensity in OPCs cultured with ACM from IL-1β, exogenous CXCL1, IL-1β + IndCl-*o*-Cl and IL-1β + IndCl-*o*-Me treated astrocytes compared to ACM alone. When ACM from IL-1β, exogenous CXCL1, IL-1β + IndCl-*o*-Cl and IL-1β + IndCl-*o*-Me treated groups was added to the cultures containing SB225002, there was no change exhibited in ACM alone, but a decrease in OL differentiation was observed in all treatment groups. n = 3 wells/group, One-Way ANOVA with Dunnett’s Multiple Comparisons Analysis and unpaired t-test.

To determine the direct effect of IndCl analogues on CXCL1 production by astrocytes and its impact on OPC survival and differentiation, we utilized primary astrocyte and OPC/OL cultures. Primary astrocytes from postnatal day 0–4 pups were isolated and treated with 13 ng/mL IL-1β, which induces astrocytic CXCL1 production^11,35^, or 13 ng/mL IL-1β concurrently with 10 nM IndCl-*o*-Cl or IndCl-*o*-Me for 48 hours. After stimulation, CXCL1 concentration was quantified in supernatant (astrocyte conditioned media; ACM) by ELISA (Fig. 6A). Astrocytes treated with IL-1β, IL-1β + IndCl-*o*-Cl and IL-1β + IndCl-*o*-Me significantly increased CXCL1 production compared to untreated astrocytes (Fig. 6A). 10 ng/mL exogenous CXCL1, which has been shown to increase OPC differentiation and survival *in vitro*^37^, was added to primary OPC cultures as a positive control. Exogenous CXCL1 treated OPCs/OLs and those treated with ACM from IL-1β, IL-1β + IndCl-*o*-Cl and IL-1β + IndCl-*o*-Me groups significantly increased MBP expression compared to ACM alone. To test whether CXCL1 mediated this phenomenon, CXCR2, the high affinity receptor for CXCL1 which is expressed by OL lineage cells, was blocked by the selective CXCR2 antagonist, SB225002^38^. CXCR2 antagonism significantly decreased MBP staining intensity in all groups. Total number of DAPI^+^ nuclei were unaffected by the addition of SB225002 in all groups aside from those that received ACM from IL-1β only-treated astrocytes (Fig. 6B–D).

### IndCl analogues increase mature OL numbers and restore myelination in callosal white matter tracts in EAE mice

IndCl and other ERβ ligands have been shown to increase white matter and subventricular zone OPC/OL populations and enhance callosal myelination in translational models of MS^10,20,29^. To test whether IndCl analogues promote similar gains in mature OL numbers and myelination, callosal white matter tracts were assessed for adenomatous polyposis coli (CC1) and MBP immunoreactivity, respectively. Additionally, ultrastructural analysis of the CC was performed by EM imaging to confirm the integrity of axon myelination.

#### Mature OLs

**Figure 7:**
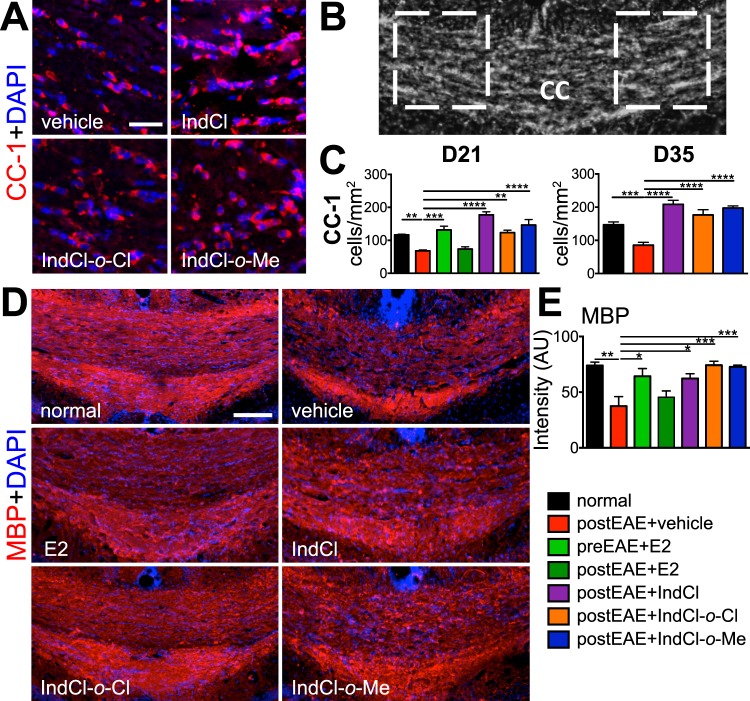
Therapeutic treatment with IndCl analogues improves the number of mature oligodendrocytes and increases myelin intensity. Mature OLs numbers **(A–C)** and myelination levels **(D-E)** were assessed by staining for adenomatous polyposis coli (CC1; red) and myelin basic protein (myelin; red) from day 21 and day 35 postEAE groups. White-dashed boxes within the normal CC **(B)**, which depict areas examined at 40X magnification in **(A)**, reveal a significant increase in numbers of mature OLs (red) in therapeutic IndCl, IndCl-*o*-Cl and IndCl-*o*-Me treated EAE mice at both time points **(C)**. **(D**-**E)** To assess myelination levels within the CC, representative 10X magnification images of midline-crossing CC from coronal brain sections (from Fig. 2A set) stained for MBP; red is shown **(D)**. All treatments, except therapeutic E2, improved MBP^+^ intensity **(E)**. n = 8 mice/group, One-Way ANOVA with Dunnett’s Multiple Comparisons Analysis. Scale bar represents 10 µM for A and 100 µM for D.

Vehicle-treated mice exhibited significant loss of CC1^+^ mature OLs relative to normal mice. Prophylactic E2 treatment did not show a decrease in CC1 cells; however, E2 treatment after disease induction was unable to rescue the decrease in CC1 cells. By contrast, therapeutic treatment with IndCl and IndCl analogues, either on the onset of disease or during peak disease, rescued the loss of CC1^+^ cells observed in vehicle-treated mice (Fig. 7A,C, SI appendix Fig. S4).

#### *MBP*^+^*myelination*

Corresponding with loss of CC1^+^ mature OLs, MBP^+^ staining was decreased in vehicle-treated mice relative to control. The presence of prophylactic E2 prevented the EAE-induced decrease in MBP^+^ staining, while therapeutic E2 was unable to rescue the decrease in MBP staining intensity as seen in vehicle-treated EAE CC (from Fig. 2A set). Also, consistent with CC1 data, IndCl, IndCl-*o*-Cl, and IndCl-*o*-Me treatment all resulted in increased MBP^+^ staining with respect to vehicle (Fig. 7D,E).

#### EM analysis

**Figure 8:**
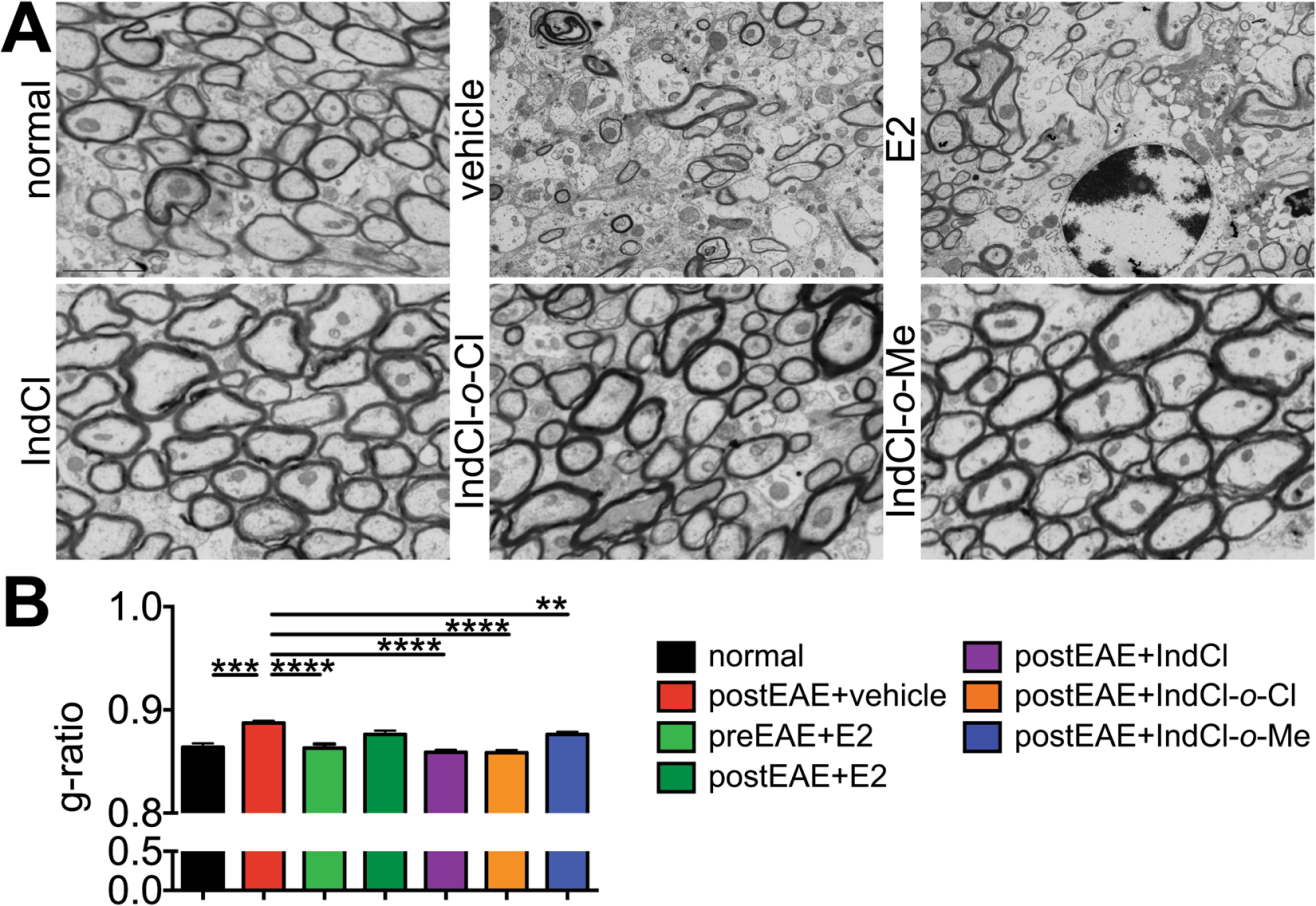
Improvement of axon myelination in IndCl analogues-treated EAE corpus callosum. **(A)** Representative electron micrographs of CC axons imaged at 14,000× magnification (brains from groups of mice from Fig. 2A set), **(B)** Prophylactic E2 reduced g-ratio relative to the vehicle-treatment level; however, therapeutic E2 did not decrease g-ratio significantly as compared to vehicle-treated group. Treatment with IndCl or either analogue, by contrast, decreased both non-myelinated axons numbers and g-ratio relative to vehicle. A minimum of 500 axons were measured per mouse. Scale bar represents 1 µM. n = 4–8 mice/group, One-Way ANOVA with Dunnett’s Multiple Comparisons Analysis.

Within a given field imaged, g-ratios were calculated by comparing mean ratio of inner axonal diameter to total outer diameter for all myelinated and non-myelinated fibers in the CC of groups of mice from Fig. 2A only. Roughly 50% of callosal fibers were non-myelinated or thinly myelinated in vehicle-treated mice compared to 10% in normal, resulting in a g-ratio that was significantly increased in vehicle-treated mice^18,20^. Prophylactic E2 reduced g-ratio relative to the vehicle-treatment level; however, therapeutic E2 did not decrease g-ratio significantly as compared to vehicle-treated group. Treatment with IndCl or either analogue, by contrast, decreased both non-myelinated axons numbers and g-ratio relative to vehicle (Fig. 8A,B).

### IndCl analogues improve fast and slow components of commissural axon conduction during EAE

**Figure 9:**
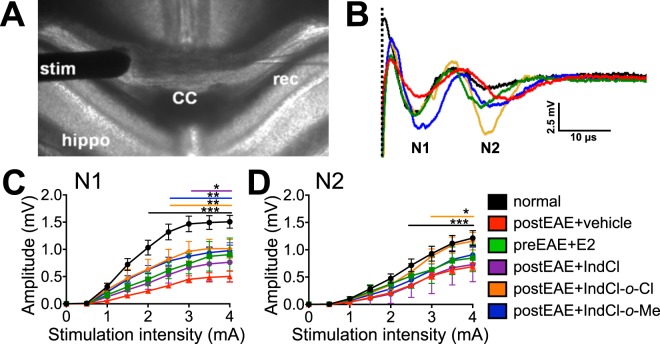
Treatment with novel estrogen receptor beta ligands increases EAE-induced callosal conduction. **(A)** Compound action potentials (CAPs) were recorded across the corpus callosum (CC) in caudal brain slices (350-µm thick) plate 48–55 (Paxinos and Franklin atlas, 2004^41^) containing the hippocampus (hippo). A recording electrode (rec) was placed 1 mm away from a bipolar stimulating electrode (stim), and voltage traces were recorded with increasing current stimulus of 0–4 mA in steps of 0.5 mA. **(B)** Voltage traces acquired with 4 mA stimulation intensity from normal (black), 30 days postEAE + vehicle (red), preEAE + E2 (green), postEAE + IndCl-*o*-Cl (orange), postEAE + IndCl-*o*-Me (blue), and postEAE + IndCl, (purple) brain slices (from Fig. 2A set). Dashed line indicates the end of the stimulus artifact and the beginning of the CAPs. The faster myelinated axon peak is indicated by “N1”, and the CAP component “N2” denotes the slower partially myelinated or unmyelinated axons peak. N1 **(C)** and N2 **(D)** CAP amplitudes of callosal axons recorded from vehicle-treated EAE slices (red) were significantly smaller than in normal controls (black). Similar to IndCl (purple), IndCl-*o*-Cl (orange) and IndCl-*o*-Me (blue) treatment resulted in improved N1. N2 amplitudes of Ind-*o*-Cl had a small but significant increase as compared to postEAE + vehicle group. n = 6–12 animals per group, Two-Way ANOVA with post hoc tests using Tukey’s multiple comparison test.

Large white matter tracts, such as the CC, are especially vulnerable to demyelination and axonal damage in MS and EAE^18,39^. Compound action potential (CAP) recordings are a valuable technique for assessing demyelination and damage in these areas through their impact on functional conductivity^19,20,40^. Thus, callosal CAPs were recorded from from Fig. 2A set-normal, IndCl, pre-E2, IndCl-*o*-Cl and IndCl-*o-*Me-treated mice brain slices corresponding approximately to plates 29–48 in the atlas of Franklin and Paxinos (2004)^41^ (Fig. 9). N1 and N2 peak amplitudes (representing fast myelinated and slower un/partially myelinated fibers, respectively) were reduced in slices from vehicle-treated mice compared to normal but were not affected by prophylactic or therapeutic E2 treatment (Fig. 9B–D). Slices from IndCl and analogue-treated mice showed significant improvement in N1 amplitude, but only IndCl-*o*-Cl treatment also increased N2 amplitude (Fig. 9B–D).

## Discussion

In search of therapeutic agents capable of reversing the progression of MS, we discovered that IndCl, a novel highly selective ERβ ligand, reduces CNS inflammation, promotes remyelination, and ameliorates disease in the EAE and cuprizone models of MS^10^. These findings prompted the current study, in which we sought to identify IndCl analogues optimized for these functions and to investigate the mechanism of their activity in greater detail. To do so, we examined the therapeutic efficacy of seven analogues of IndCl with single substitutions to the phenol ring (and one that was di-substituted), each of which retained selectivity for ERβ binding over ERα. After an initial OPC differentiation screening assay, we found that three analogues, IndCl-*o*-Cl, IndCl-*o*-Me, and IndCl-*o*-I had activity equivalent to that of IndCl itself, and from these, we selected the best two, IndCl-*o*-Cl and IndCl-*o*-Me, for subsequent in-depth evaluation of their effects in mice with EAE. These studies found that the IndCl analogues tested shared many therapeutic qualities in common with their parent compound, but also displayed several unique benefits not observed with IndCl treatment that speak to their promise for ultimate clinical utility (a summary of which can be found in Table 1**)**. In these studies, none of the chloroindazole compounds demonstrated any apparent cellular or *in vivo* toxicities, while structurally they all conform to a pharmacophore model typical for ERβ-selective ligands^13,15^. IndCl itself has been extensively studied in cellular and *in vivo* models of endometriosis and was found to have good, ERβ-dependent efficacy with no apparent toxicities^16^.

**Table 1:** Summary of *in vivo* E2, IndCl, and IndCl analogue treatment effects compared to vehicle.

Category	Marker	Pre E2	Post E2	IndCl	IndCl-*o*-Cl	IndCl-*o*-Me
Disability	Disease score^§^	−	°	°, -	−	−
	Rotarod performance^§^	+	°	°	+	+
Myelination	OLs	+	+	+	+	+
	MBP	+	°	+	+	+
	g-ratio	−	°	−	−	−
Peripheral Inflammatory Cytokines	IFN*γ*	−	−	−	−	−
	IL-1β^§^	°	°	+	°	°
	IL-2	−	°	°	°	°
	IL-6^§^	−	−	°	−	−
	IL-17^§^	−	−	°	−	−
	TNFα	°	°	°	°	°
Peripheral Th2 & Anti-inflammatory Cytokines	IL-4	°	°	°	°	°
	IL-5	°	°	°	°	°
	IL-10	°	°	°	°	°
	IL-13^§^	°	°	°	−	−
Peripheral Chemokines	CXCL1	+	+	+	+	+
	CXCL10	−	−	−	−	−
CNS Chemokines	CXCL1	°	+	+	+	+
CNS Cellular Inflammation	CD45	−	−	−	−	−
	GFAP	−	°	°	°	°
Callosal CAP Electrophysiology	N1 amplitude	°	°	+	+	+
	N2 amplitude^§,#^	°	°	°	+	°

Summary of experimental results obtained from EAE mice treated prophylactically with E2 or therapeutically with E2, IndCl, or IndCl analogues organized by category and marker assayed. Cells containing. ‘+’ indicate marker was significantly (p ≤ 0.05) increased in treatment group relative to vehicle. ‘−’ indicates marker was significantly decreased in treatment group relative to vehicle; and ‘o’ indicates no change as compared to vehicle treated groups; ^§^adjacent to a marker indicates differential performance between IndCl and one or more IndCl analogue. ^#^Indicates differential performance between analogues.

### Common effects of IndCl and analogues

Consistent with past reports, treatment with IndCl or either of the two analogues tested improved parameters related to myelination and inflammation with respect to vehicle alone^10,12^. Examination of thoracic dorsal horn and CC white matter showed increased myelination and greater numbers of mature callosal OLs in these groups, suggesting myelination induced by IndCl or its analogues was a product of preserving or replenishing OL populations. While the precise mechanism whereby IndCl analogues promote these changes remains uncertain, previous studies have found that IndCl and another ERβ ligand, diarylpropionitrile, produced similar effects on myelination through induction of PI3K/Akt/mTOR signaling^10,14,27^.

Importantly, these findings corresponded with improvement in functional measures of myelin recovery. Ultrastructural analysis of callosal white matter revealed greater numbers of myelinated fibers and thicker myelin sheaths overall, indicating that IndCl and analogue-induced myelin production was correctly targeted to axons. Similarly, CAP recordings from these groups demonstrated improvement in N1 peak amplitude, suggesting more myelinated axons or a larger response from those present in the evoked fiber volley^22,42^. Consistent with previous reports examining ERβ ligands in EAE, these benefits were observed in the presence of ongoing cellular inflammation, denoted by the lack of effect on astrogliosis or leukocyte infiltration.

Examination of cytokines secreted by splenic leukocytes from these animals suggested this maybe partially due to suppression of IFN*γ* and CXCL10 production, both of which are potent mediators of OL death^34,43,44^. IFNγ is a major pro-inflammatory cytokine and is found in MS lesions as well as in activated blood mononuclear cells in progressive MS patients^45,46^. However, IFNγ may have a protective role in late EAE by regulating myelin debris removal by CNS antigen presenting cells^47^. Furthermore, low levels of IFNγ protected cultured OLs against oxidative stress, thus preventing their death^48^. IFNγ was significantly decreased by all ERβ ligands compared to vehicle, suggesting a role for these ligands in protecting OLs. CXCL10, also known as Interferon gamma-induced protein 10 (IP-10), an IFNγ dependent chemoattractant for T lymphocytes, is upregulated in the cerebrospinal fluid and CNS lesions of MS patients^49^. Similar to what is observed with E2 and IndCl treatment during EAE, antibody-mediated systemic blockade of CXCL10 signaling has been reported to prevent recruitment of activated CD4^+^ T cells and diminished EAE severity^50^. IndCl and analogues significantly decreased CXCL10 levels in the periphery. Additionally, the induction of CXCL1 both *in vivo* within the periphery and CNS of mice treated with IndCl or its analogues as well as *in vitro* may play a role in promoting the pro-myelinating effects observed. Interestingly, CXCL1 upregulation in the spinal cord was noted in mice that received IndCl or analogue treatment, but not E2, suggesting that this effect may be antagonized by ERα signaling^11^.

Although best known as a neutrophil chemoattractant^51^, astrocyte-derived CXCL1 signaling through its receptor, CXCR2, on OPCs is essential for normal developmental myelination^52,53^. Several lines of evidence suggest CXCL1 may be harnessed for its therapeutic potential in the adult CNS. CXCL1^+^ astrocytes and CXCR2^+^ OPCs have been noted at the borders of active, but not silent, MS lesions where spontaneous myelination has been documented^35^. Additionally, CXCL1 contributes to OPC proliferation and migration^37^, and CXCR2 signaling protects OPCs from IFN*γ* and CXCL10-induced apoptosis by increasing levels of the anti-apoptotic protein, Bcl-2 *in vitro*^34^. CXCL1 overexpression by GFAP^+^ astrocytes ameliorate EAE disease severity during late disease (day 30 onwards), similar to what is observed with ERβ ligand treatment^54^. IL-1β is associated with the pathophysiology of various inflammatory and demyelinating disorders^55,56^. Although IL-1β has been shown to be cytotoxic to mature OLs *in vitro*, it is crucial in CNS repair, as IL-1β−/− mice fail to remyelinate properly, possibly through the induction of astrocyte and microglia–macrophage-derived insulin growth factor-1^57^. We have demonstrated that ACM from IL-1β treated cultures induced CXCL1 expression which promoted OPC differentiation to MBP^+^ OLs. When CXCR2 is blocked with SB225002, we observed significant OL death, suggesting the importance of CXCR2 in promoting OL survival and differentiation, as previously demonstrated^34^. However, when IndCl analogues were added in combination with IL-1β treatment, there was no significant difference in the number of OLs in the presence nor absence of SB225002, although MBP intensity is significantly reduced with SB225002 treatment. These results suggest that besides increasing CXCL1 production, which enhances OPC recruitment and differentiation, ERβ ligands may also skew the proinflammatory environment to one associated with myelin repair by promoting OL survival and myelination.

Together, these findings suggest that IndCl-based compounds stimulate functional remyelination by altering inflammatory responses associated with OL apoptosis, while upregulating cytokine programs involved in developmental myelination. Future studies will address whether the results described above represent lynchpins of the pro-myelinating functions of IndCl-family molecules and whether additional factors play a role in their therapeutic effects.

### Differential effects between IndCl, IndCl-*o*-Cl, and IndCl-*o*-Me

While both IndCl and its two analogues improved myelination and modulated cytokine production associated with both demyelination and remyelination, key differences emerged in their impact on neurological disability, cytokine milieu, and electrophysiological measures. In contrast with previous reports, only IndCl*-o-*Cl and IndCl*-o-*Me reduced clinical disease severity, while IndCl did not significantly alter clinical disability (Fig. 2A)^10,11^. Although it is unclear why IndCl performed differently, one source of variation comes from the earlier time point at which treatments were initiated in the present study. This may arise from IndCl’s weak effect on leukocyte infiltration and CNS cytokine production. Earlier studies, initiated treatment during peak or chronic EAE, which features ongoing inflammatory leukocyte infiltration, but reduced cytokine production^58^. Initiation of IndCl therapy during the acute phase of EAE may have been less effective at attenuating the prolific production of inflammatory cytokines characteristic of this time point^58^. However, Ind-Cl, similar to IndCl-*o*-Cl and IndCl-*o*-Me, reduced clinical disease severity when they were administered during peak disease (Fig. 2B), Additional study is required to determine how ERβ ligand signaling alters disease kinetics at earlier versus later stages of EAE.

Related to their effect on clinical disease, IndCl*-o-*Cl and IndCl*-o-*Me reduced production of cytokines related to Th17 differentiation. Th17 cells represent a CD4+ T cell population that are induced and activated by exposure to IL-1β, IL-6, IL-23, and transforming growth factor β^59^. In EAE and MS, Th17 cells exacerbate blood-brain barrier permeability, demyelination, and axon damage through release of factors that potentiate the cytotoxic properties of ongoing inflammatory processes^60^. Both IndCl analogues reduced peripheral IL-6 production, potentially contributing to the decreased IL-17 also observed with analogue treatment. No such decrease was seen in either parameter with IndCl. Given the much greater selectivity for ERβ over ERα exhibited by IndCl, it is possible this reflects weak partial ERα agonism by the analogues tested, due to their somewhat reduced ERβ binding selectivity (Fig. 1A). Interestingly, IndCl treatment has shown to increase peripheral IL-1β production which we have previously shown is important for CXCL1 production and has a positive effect on myelination and immunomodulation^11^.

In addition to reducing peripheral Th1 and Th17 cytokines, IndCl*-o-*Cl and IndCl*-o-*Me had the unexpected effect of also suppressing peripheral production of the Th2 cytokine, IL-13. Driving Th2 polarization is protective in EAE and MS, and elevated cerebrospinal fluid concentrations of IL-13 correlate with improved measures of neuronal integrity and cortical inhibition in MS patients in patients with MS. However, IL-13 also upregulates major histocompatibility complex II on monocytes, and global IL-13 knockout lowers susceptibility to EAE in female mice. Thus, the consequences of its reduction in the current study warrant further investigation.

Among the IndCl-related compounds studied, IndCl*-o-*Cl displayed a potential benefit not observed with other treatments. Callosal CAP recordings revealed that in addition to improving the fast, myelinated component, IndCl*-o-*Cl also rescued slower conduction by small, unmyelinated, or partially myelinated fibers. As lower motor neuron loss and reduction of remaining neurites is a feature of similar EAE paradigms, this result suggests IndCl*-o-*Cl may exert neuroprotective effects that outstrip the other IndCl-based molecules included in this study.

Through our examination of the functional, histopathological, and immunological basis of the pro-myelinating effects of IndCl-based ERβ ligands, we have shown that two of the IndCl analogues tested exhibit therapeutic benefits exceeding their parent compound. While treatment with IndCl, IndCl-*o*-Cl and IndCl-*o*-Me resulted in enhanced myelination, IndCl-*o*-Cl and IndCl-*o*-Me improved neurological outcomes and suppressed inflammatory cytokine production better than their parent compound. Further support that modification of the base IndCl molecule differentially effects its impact on demyelinating disease is evidenced by IndCl-*o*-Cl uniquely demonstrating support of unmyelinated axon health in the form of improved N2 amplitude. Thus, IndCl itself, but even more so the two analogues IndCl-*o*-Me and IndCl-*o*-Cl, represent a class of ERβ ligands that offer potent remyelination and neuroprotection as well as modulation of the immune system that may be fine-tuned by additional refinement and substitution. The lack of any discernable side effects for the compounds we have thus far studied, and in other work for IndCl itself, is also of note^16^. For these reasons, this family of molecules appear appropriate to consider for further therapeutic development in the treatment of MS and other diseases affecting myelination and neurodegeneration.

## Supplementary information

Supporting Information

## Data Availability

All data generated or analyzed during this study are included herein and in the *Supporting Information appendix*.
