# Supplementary material for: Analogues of ERβ ligand chloroindazole exert immunomodulatory and remyelinating effects in a mouse model of multiple sclerosis
Source: Sci Rep. 2019 Jan 24;9:503. doi: 10.1038/s41598-018-37420-x (PMC6345788; doi:10.1038/s41598-018-37420-x)
Supplement: Supplementary file 1 — Supporting Information [file 41598_2018_37420_MOESM1_ESM.pdf]

Supporting Information

Title: Analogues of ERβ ligand chloroindazole exert immunomodulatory and remyelinating effects in a mouse model of multiple sclerosis

Authors: Hawra Karim[1#], Sung Hoon Kim[2#], Kelli Lauderdale[1], Andrew S. Lapato[1], Kelley Atkinson[1], Norio Yasui[2], Hana Yamate-Morgan[1], Maria Sekyi[1], John A. Katzenellenbogen[2], and Seema K. Tiwari-Woodruff[1, 3*]

1. Division of Biomedical Sciences, UCR School of Medicine, Riverside, CA 92521

2. Department of Chemistry, University of Illinois at Urbana-Champaign, Urbana, IL 61801

3. Center for Glia Neuronal Interaction, UCR School of Medicine, Riverside, CA 92521

Equal first authors

Corresponding Author

*Seema K. Tiwari-Woodruff, Ph.D.
Division of Biomedical Sciences
School of Medicine, University of California Riverside
Room 205, 311 School of Medicine Research Building
900 University Ave, Riverside, CA 92521, USA
Work No.: (951) 827-7819
Email: seema.tiwari-woodruff@medsch.ucr.edu; seema-tiwari-woodruff@axonremyelination.org

TABLE OF CONTENTS PAGE

A. Synthesis and spectroscopic characterization of new IndCl analogs.

Synthetic Scheme ………………………………………………………………... **3**

Experimental Procedures and Spectroscopic Information……………………..…. **4-10**

Estrogen Receptor Binding Affinity Assays ……………………………………**10**

B. Supplementary IHC Figures ………………………………………………… 11-14

C. Detailed Materials and Methods……………………………………………… 15-20

D. References…………………………………………………………………21

A. Synthesis and spectroscopic characterization of new IndCl analogs.

General Synthetic Scheme for the Synthesis of IndCl Analogs.

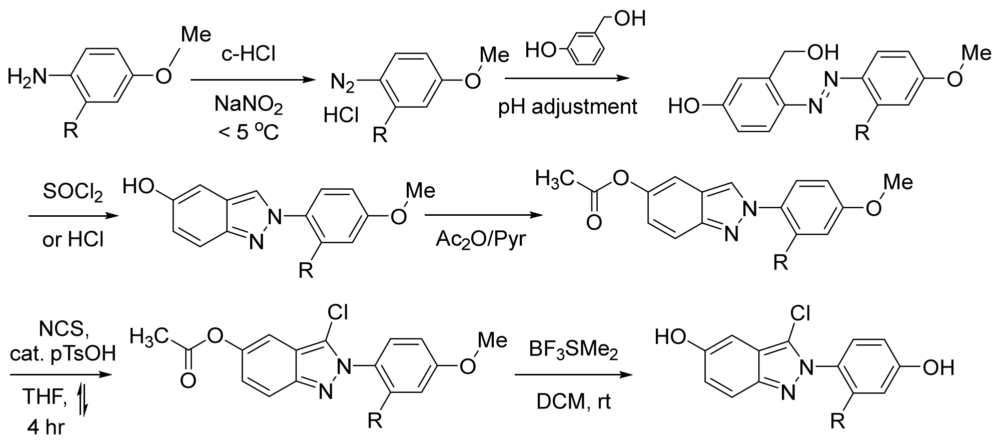

Synthetic Methods

Synthesis of 3-chloro-2-(2-chloro-4-hydroxyphenyl)-2H-indazol-5-ol (IndCl-*o*-Cl):

1. 2-(2-chloro-4-methoxyphenyl)-2H-indazol-5-ol

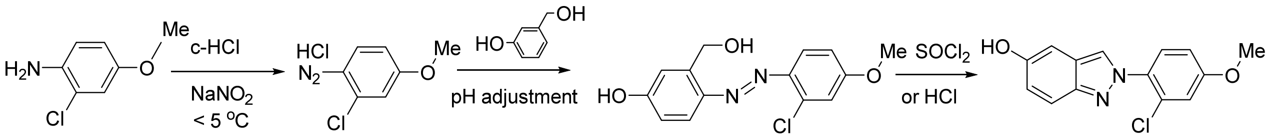

To the suspension of 2-chloro-4-methoxyaniline (628 mg, 4.00 mmol) in cold DI water (10 mL) was added *c*-HCl (1.2 mL) and subsequently sodium nitrite (284 mg, 4.11 mmol) added portionwise a in ice bath by maintaining the temperature below 5 ºC. After 1.5 hr stirring of the reaction mixture at below 5 ºC, 3-hydroxymethylphenol (496 mg, 4.00 mmol) in a water-acetone (1:1, v/v, 5 ml) mixture was added into the reaction mixture in ice bath, followed by adjusting the pH up to 7.5 with 1N NaOH to precipitate a yellowish solid. During the addition of the NaOH aq. solution, a massive yellowish solid precipitated from solution as the pH reached ~7.5.

To form the Indazole ring: Method 1) the solid was collected by filtration, washed with DI water, and dried in an oven at 60 ~80 ºC (1.1 g yellowish solid). (It is not necessary to adjust pH acidic in this case to collect a solid, but in some case the pH of the solution needs to be acidifies to collect more solid.). The collected solid used without further purification.

^{1}HNMR (500 MHz, CD_3OD-$CDCl_3$) δ 3.83 (s, 3H), 4.92 (s, 2H), 6.81 (dd, J = 8.5, 3.0 Hz, 1H), 6.83 (dd, J = 8.5, 3.0 Hz, 1H), 6.93 (d, J = 3.0 Hz, 1H), 7.01 (d, J = 3.0 Hz, 1H), 7.63 (d, J = 8.5 Hz, 1H), 7.77 (d, J = 8.5 Hz, 1H). ^{13}CNMR (126 MHz, CD_3OD-$CDCl_3$) δ 55.93, 62.04, 114.09, 114.95, 115.17, 115.37, 118.75, 123.40, 136.35, 139.98, 143.19, 143.99, 160.52, 161.86.

The dried yellowish solid was suspended into 20 mL dichlomethane and treated with a thionyl chloride (2.00 g, 16.94 mmol) for 30 min in an ice bath. Once thionyl chloride was added, the solution was turned into scarlet color and of the all suspended solid dissolved. To the reaction mixture was added a powder of sodium bicarbonate (3.50 g, 41.67 mmol) and dropwise ice-water, until most of sodium bicarbonate was gone. The precipitate that formed was collected by filtration (710 mg).

^{1}HNMR (500 MHz, CD_3OD-$CDCl_3$) δ 3.82 (s, 3H), 6.88 (dd, J = 2.5, 8.5 Hz, 1H), 6.90 (s, 1H), 7.00 (dd, J = 8.5, 2.5 Hz, 1H), 7.02 (d, J = 2.5 Hz, 1H), 7.44 (d, J = 8.5 Hz, 1H), 7.55 (d, J = 8.5

Hz, 1H), 7.96 (s, 1H). ^{13}CNMR (126 MHz, CD_3OD-$CDCl_3$) δ 59.97, 103.96, 117.47, 119.57, 122.53, 125.84, 126.43, 128.40, 133.17, 134.48, 135.66, 149.90, 156.11, 164.44.

Method 2) The diazo compound in aqueous solution was extracted with ethyl acetate (50 mL x 3). To the extract was added 1 mL of c-HCl, and the resulting solution was refluxed until the yellowish diazo compound disappeared on silica gel TLC (20 % ethyl acetate in n-hexane, v/v, Rf ~0.8) and new blue fluorescent spot (Rf ~0.65) appeared. The reaction mixture was washed with sat. aq. sodium bicarbonate solution, brine, water, dried over sodium sulfate, followed by filtration and evaporation to afford an enough pure title compound (680 mg) as a pale brownish solid.

2. *Synthesis of 3-chloro-2-(2-chloro-4-methoxyphenyl)-2H-indazol-5-yl acetate*

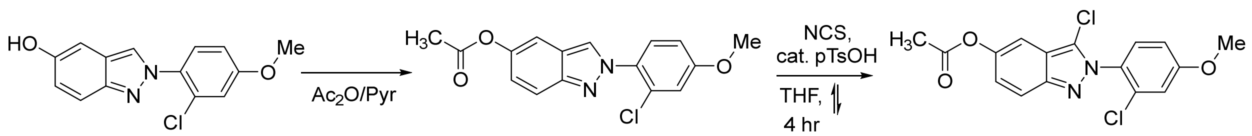

To the powder of 2-(2-chloro-4-methoxyphenyl)-2H-indazol-5-ol (400 mg, 1.46 mmol) was added of acetic anhydride (2 mL) and dry pyridine (1 mL). The resultant solution was heated up 90 °C for 1 hr, and evaporation afforded a 2-(2-chloro-4-methoxyphenyl)-2H-indazol-5-yl acetate (455 mg).

^{1}HNMR (500 MHz, $CDCl_3$) δ 2.36 (s, 3H), 3.90 (s, 3H), 6.97 (dd, J = 3.0, 8.5 Hz, 1H), 7.08 (dd, J = 8.5, 3.0 Hz, 1H), 7.10 (d, J = 3.0 Hz, 1H), 7.44 (d, J = 3.0 Hz, 1H), 7.56 (d, J = 8.5 Hz, 1H), 7.80 (d, J = 8.5 Hz, 1H), 8.24 (s, 1H). ^{13}CNMR (126 MHz, $CDCl_3$) δ 21.45, 56.17, 111.26, 113.73, 115.68, 119.53, 121.56, 123.22, 126.07, 129.36, 130.32, 131.93, 145.97, 147.70, 160.56, 170.27.

To the acetyl compound (184 mg, 0.582 mmol) in THF (5 mL) was added NCS (80 mg, 0.60 mmol) and cat. amount of *p*-TsOH, and the resulting solution was refluxed for 4 hr, allowed to cool down to room temperature, passed through short silica gel column (2.5 X 3 cm) with 30 % ethyl acetate in n-hexane (v/v) to afford a 3-chloro-2-(2-chloro-4-methoxyphenyl)-2H-indazol-5-yl acetate (193 mg) as a colorless solid. Over the time of the chlorination, the blue fluorescent starting material spot was slowly disappeared on silica gel TLC and slightly higher Rf value of non-blue fluorescent spot was appeared on TLC.

^{1}HNMR (500 MHz, $CDCl_3$) δ 2.38 (s, 3H), 3.93 (s, 3H), 6.99 (dd, J = 2.5, 8.5 Hz, 1H), 7.12 (dd, J = 2.5, 8.5 Hz, 1H), 7.14 (d, J = 3.0 Hz, 1H), 7.38 (d, J = 3.0 Hz, 1H), 7.42 (d, J = 8.5 Hz, 1H),

7.74 (d, J = 8.5 Hz, 1H). ^{13}CNMR (126 MHz, $CDCl_3$) δ 21.41, 56.19, 110.04, 113.58, 115.54, 118.52, 120.15, 123.45, 124.28, 128.65, 130.23, 133.28, 146.31, 147.23, 161.54, 170.14.

Synthesis of 3-chloro-2-(2-chloro-4-hydroxyphenyl)-2H-indazol-5-ol (IndCl-o-Cl)

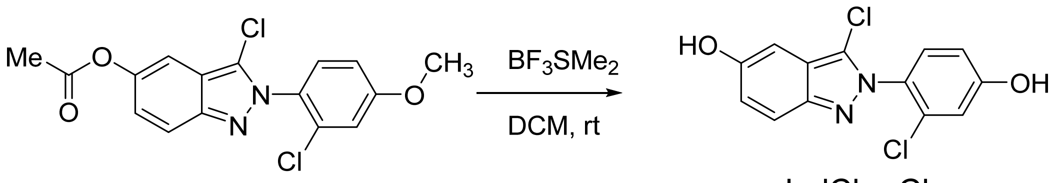

IndCl-*o*-Cl

3-Chloro-2-(2-chloro-4-methoxyphenyl)-2H-indazol-5-yl acetate (193 mg, 0.50 mmol) was dissolved into dichloromethane (5 ml). To the resulting solution boron trifluoride dimethyl sulfide (700 mg, 5.38 mmol) was added and the reaction vessel was stirred for 4 hr at rt. Once the starting material had disappeared upon silica gel TLC analysis, the solvent and excess the boron trifluoride dimethyl sulfide were evaporated. DI water (5 mL) was added to the residue, and the mixture was sonicated for 20 min to form ppt, followed by filtration to collect the colorless solid and dried to afford the title compound (IndCl-*o*-Cl) (162 mg).

^{1}HNMR (500 MHz, CD_3OD-$CDCl_3$) δ 6.76 (d, J = 3.0 Hz, 1H), 6.79 (dd, J = 2.5, 8.5 Hz, 1H), 6.95 (d, J = 2.5 Hz, 1H), 7.00 (dd, J = 2.5, 8.5 Hz, 1H), 7.21 (d, J = 8.5 Hz, 1H), 7.47 (d, J = 8.5 Hz, 1H). ^{13}CNMR (126 MHz, CD_3OD-$CDCl_3$) δ 98.41, 114.77, 116.94, 119.08, 119.23, 120.88, 122.99, 127.51, 130.12, 132.95, 145.23, 152.64, 159.67.

HRMS (ESI, M^{+}+1) $C_{13}H_9Cl_2N_2O_2$ Calcd. 295.0041, found 295.0051.

Synthesis of 3-chloro-2-(4-hydroxy-2-methylphenyl)-2H-indazol-5-ol (IndCl-*o*-Me)

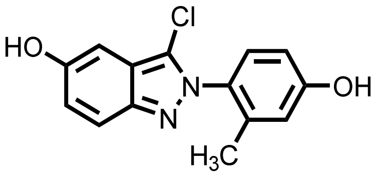

IndCl-*o*-Me

IndCl-*o*-Me was obtained from the reaction of 2-methyl-4-methoxyaniline (411 mg, 3.00 mmol) with 3-hydroxymethylphenol (397 mg, 3.20 mmol) in 63 % yield through 5 steps, following the same procedure described for the preparation of IndCl-*o*-Cl.

^{1}HNMR (500 MHz, CD_3OD-$CDCl_3$) δ 1.93 (s, 3H), 6.73 (dd, *J* = 2.5, 8.5 Hz, 1H), 6.76 (d, *J* = 2.5 Hz, 1H), 6.78 (d, *J* = 2.5 Hz, 1H), 7.01 (dd, *J* = 2.5, 8.5 Hz, 1H), 7.10 (d, *J* = 8.5 Hz, 1H), 7.50 (d, *J* = 8.5 Hz, 1H). ^{13}CNMR (126 MHz, CD_3OD-$CDCl_3$) δ 17.09, 98.36, 113.44, 117.27, 118.87, 119.24, 120.15, 122.57, 128.82, 129.28, 137.43, 144.84, 152.69, 158.69.

HRMS (ESI, M^{+}+1) $C_{14}H_{12}ClN_2O_2$ Calcd. 275.0587, found 275.0587.

Synthesis of 3-chloro-2-(4-hydroxy-2-(trifluoromethyl)phenyl)-2H-indazol-5-ol (IndCl-*o*-CF_3)

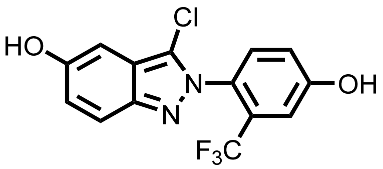

IndCl-*o*-CF_3

IndCl-*o*-CF_3 was prepared from the reaction of 2-trifluoromethyl-4-methoxyaniline (382 mg, 2.00 mmol) with 3-hydroxymethylphenol (272 mg, 2.20 mmol) in 71% yield through 5 steps, following the same procedure described for the preparation of IndCl-*o*-Cl.

^{1}HNMR (500 MHz, CD_3OD-$CDCl_3$) δ 6.77 (dd, *J* = 2.5, 8.5 Hz, 1H), 7.02-7.033 (m, 2H), 7.19-7.24 (m, 2H), 7.50 (dd, *J* = 2.5, 8.5 Hz, 1H). ^{19}FNMR (470 MHz, CD_3OD-$CDCl_3$) δ -61. ^{13}CNMR (126 MHz, CD_3OD-$CDCl_3$) δ 98.36, 114.46 (J = 4.6 Hz), 118.99, 119.16, 122.65 (J = 259 Hz), 123.29, 126.38, 129.26 (J = 32.38 Hz), 131.71, 140.05, 142.63, 144.85, 152.79, 159.36.

HRMS (ESI, M^{+}+1) $C_{14}H_7ClN_2O_2F_3$ Calcd. 327.0148, found 327.0143.

Synthesis of 3-chloro-2-(2-fluoro-4-hydroxyphenyl)-2H-indazol-5-ol (IndCl-*o*-F)

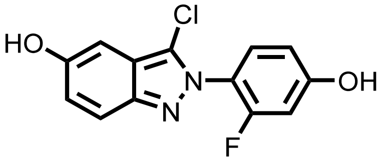

IndCl-*o*-F

IndCl-*o*-F was prepared from the reaction of 2-fluor-4-methoxyaniline (282 mg, 2.00 mmol) with 3-hydroxymethylphenol (272 mg, 2.20 mmol) in 68% yield through 5 steps, following the same procedure described for the preparation of IndCl-*o*-Cl.

^{1}HNMR (500 MHz, CD_3OD-$CDCl_3$) δ 6.67-6.72 (m, 2H), 6.77 (d, J = 2.5 Hz, 1H), 7.01 (dd, J = 2.5, 8.5 Hz, 1H), 7.25 (t, J = 9.0 Hz, 1H), 7.48 (d, J = 8.5 Hz, 1H). ^{19}FNMR (470 MHz, CD_3OD-$CDCl_3$) δ -120.09 (t, J = 38.1 Hz, 1H). ^{13}CNMR (126 MHz, CD_3OD-$CDCl_3$) δ 98.36, 103.83 (J = 22.18 Hz), 111.93, 117.65 (J = 7.4 Hz), 118.96, 119.46, 120.89, 123.10, 129.49, 145.22, 125.71, 156.81, 159.11 9J = 259.11 Hz).

HRMS (ESI, M^{+}+1) $C_{13}H_7ClN_2O_2F$ Calcd. 277.0189, found 277.0176.

Synthesis of 2-(2-bromo-4-hydroxyphenyl)-3-chloro-2H-indazol-5-ol (IndCl-*o*-Br)

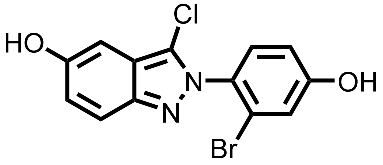

IndCl-*o*-Br

IndCl-*o*-Br was prepared from the reaction of 2-bromo-4-methoxyaniline (404 mg, 2.00 mmol) with 3-hydroxymethylphenol (272 mg, 2.20 mmol) in 56% yield through 5 steps, following the same procedure as described for the preparation of IndCl-*o*-Cl.

^{1}HNMR (500 MHz, CD_3OD-$CDCl_3$) δ 6.79 (d, *J* = 3.0 Hz, 1H), 6.84 (dd, *J* = 3.0, 8.5 Hz, 1H), 7.04 (dd, *J* = 3.0, 8.5 Hz, 1H), 7.14 (d, *J* = 3.0 Hz, 1H), 7.23 (d, *J* = 8.5 Hz, 1H), 7.51 (d, *J* = 8.5 Hz, 1H). ^{13}CNMR (126 MHz, CD_3OD-$CDCl_3$) δ 98.55, 115.38, 119.03, 119.27, 120.09, 121.11, 122.43, 123.24, 128.95, 130.13, 144.84, 152.74, 159.71.

HRMS (ESI, M^{+}+1) $C_{13}H_9ClBrN_2O_2$ Calcd. 338.9536, found 338.9549.

Synthesis of 2-(2-iodo-4-hydroxyphenyl)-3-chloro-2H-indazol-5-ol (IndCl-*o*-I)

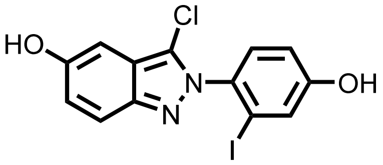

IndCl-*o*-I

IndCl-*o*-I was prepared from the reaction of 2-bromo-4-methoxyaniline (500 mg, 2.00 mmol) with 3-hydroxymethylphenol (272 mg, 2.20 mmol) in 51% yield through 5 steps, following the same procedure described for the preparation of IndCl-*o*-Cl.

^{1}HNMR (500 MHz, CD_3OD-$CDCl_3$) δ 6.78 (d, *J* = 3.0 Hz, 1H), 6.87 (dd, *J* = 3.0, 8.5 Hz, 1H), 7.02 (dd, *J* = 3.0, 8.5 Hz, 1H), 7.17 (d, *J* = 8.5 Hz, 1H), 7.37 (d, *J* = 3.0 Hz, 1H), 7.50 (d, *J* = 8.5 Hz, 1H). ^{13}CNMR (126 MHz, CD_3OD-$CDCl_3$) δ 97.07, 98.52, 116.05, 119.22, 119.44, 120.42, 123.02, 126.19, 129.36, 132.82, 145.01, 152.65, 159.26.

HRMS (ESI, M^{+}+1) $C_{13}H_9ClIN_2O_2$ Calcd. 386.9397, found 386.9379.

Synthesis of 2-(2-chloro-4-hydroxyphenyl)-3,4-dichloro-2H-indazol-5-ol (IndCl-*o*-Cl-4-Cl)

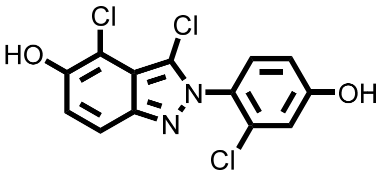

IndCl-*o*-Cl-4-Cl

IndCl-o-Cl-4-Cl was prepared from the reaction of IndCl-*o*-Cl (29.4 mg, 0.10 mmol) with NCS (13.0 mg, 0.10 mmol) in THF (1.0 mL) containing catalytic amount of p-TsOH at 45 °C in 67% (22.0 mg) yield as a pale grey solid after silica gel chromatographic purification with a mixture of methanol and dichloromethane (5:95, v/v) as an eluent.

^{1}HNMR (500 MHz, CD_3OD-$CDCl_3$) δ 6.85 (dd, J = 2.5, 9.0 Hz, 1H), 7.00 (d, J = 2.5 Hz, 1H), 7.09 (d, J = 9.0 Hz, 1H), 7.24 (d, J = 8.5 Hz, 1H), 7.41 (d, J = 8.5 Hz, 1H). ^{13}CNMR (126 MHz, CD_3OD-$CDCl_3$) δ 105.34, 114.80, 116.60, 116.91, 117.58, 121.65, 122.59, 127.24, 130.16, 132.99, 145.63, 148.49, 159.96.

HRMS (ESI, M^+-1, neg. mode) $C_{13}H_6Cl_3N_2O_2$ Calcd. 326.9495, found 326.9488.

Estrogen Receptor Binding Affinity Assays. Relative binding affinities were determined by a competitive radiometric binding assay as previously described[3]#[4] using 10 nM [^{3}H]-estradiol as tracer ([6,7-^{3}H]estra-1,3,5,(10)-triene-3,17-*β*-diol, 51-53 Ci/mmol, Amersham Biosciences, Piscataway, NJ), and purified full-length human ERα and ER*β* were purchased from PanVera (Madison, WI). Incubations were for 18-24 h at 0 °C. Hydroxyapatite (Bio-Rad, Hercules, CA) was used to absorb the receptor-ligand complexes, and free ligand was washed away. The binding affinities are expressed as relative binding affinity (RBA) values with the RBA of estradiol set to 100%. The values given are the average ± range or SD of two or three independent determinations. RBA = {IC_{50}[estradiol]/IC_{50}[compound]} x 100. Values are the mean ± range or SD of 2 or more independent experiments. The RBA for estradiol is 100; K_d value for estradiol is 0.2 nM for ERα and 0.5 nM for ERβ.

B. Supplemental Figures

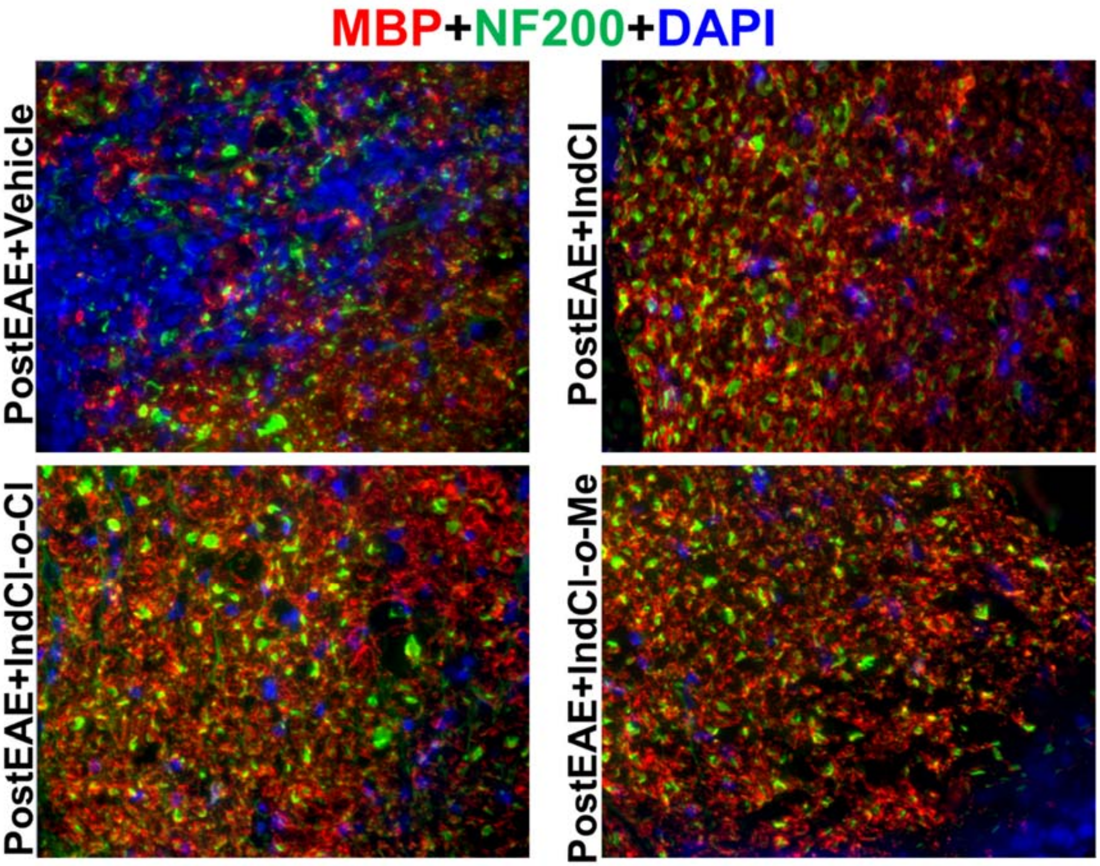

Supplementary Fig. S1. Treatment with vehicle, IndCl, IndCl-*o*-Cl, IndCl-*o*-Me was started on peak EAE disease on day 17 and continued until day 35. Mice were sacrificed and perfused with saline followed by 10% formalin at day 35 postEAE (from Figure 2B). CNS IHC was performed to assess inflammation, and myelination. Representative 40x magnification coronal images of the ventral column of thoracic spinal cord collected on day 35. Tissues from vehicle, IndCl, IndCl-*o*-Cl and IndCl-*o*-Me treated groups were immunostained with myelin basic protein (MBP; red), neurofilament 200 (NF200; green) and nuclear stain DAPI (blue).

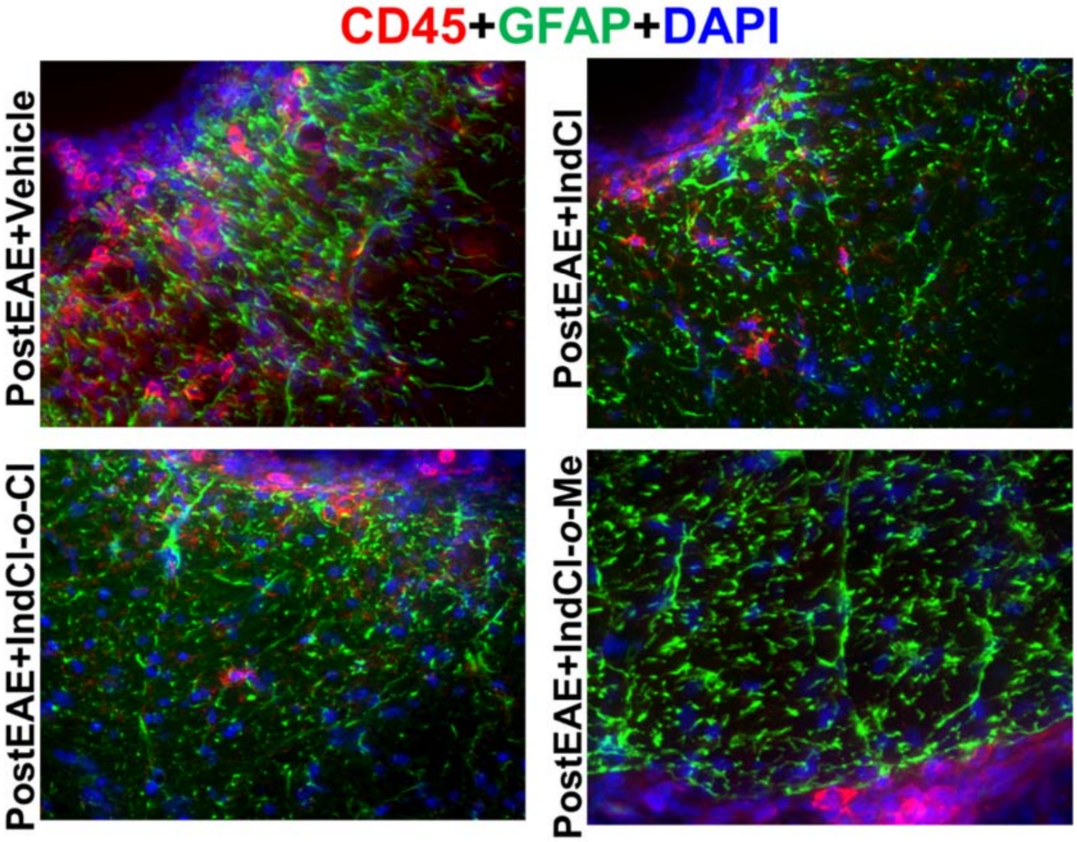

Supplementary Fig. S2. Treatment with vehicle, IndCl, IndCl-*o*-Cl, IndCl-*o*-Me was started on peak EAE disease on day 17 and continued until day 35. Mice were sacrificed and perfused with saline followed by 10% formalin at day 35 postEAE (from Figure 2B). CNS IHC was performed to assess inflammation, and myelination. Representative 40x magnification images of the spinal cord dorsal column from day 35 postEAE treated with vehicle, IndCl, IndCl-*o*-Cl and IndCl-*o*-Me were immunostained with cluster of differentiation (CD)45 (red) and glial fibrillary acidic protein (GFAP; green), and DAPI (blue).

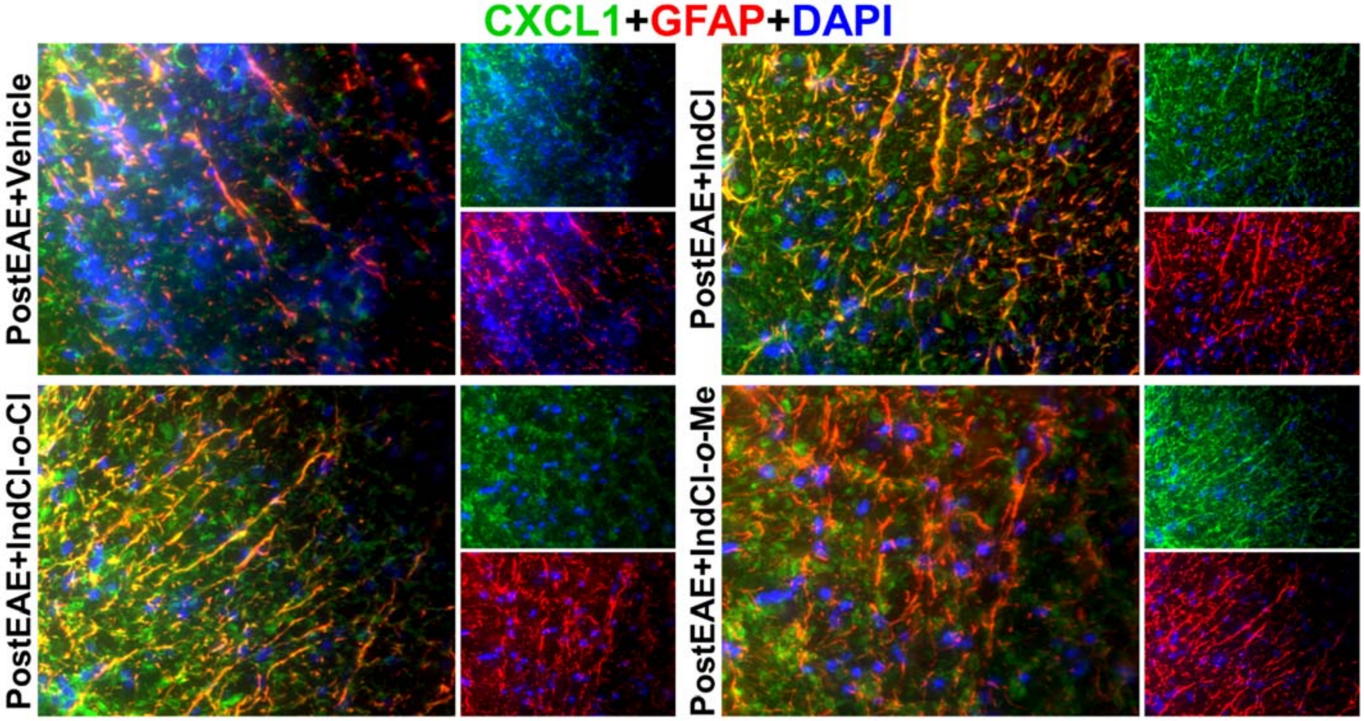

Supplementary Fig. S3. Treatment with vehicle, IndCl, IndCl-*o*-Cl, IndCl-*o*-Me was started on peak EAE disease on day 17 and continued until day 35. Mice were sacrificed and perfused with saline followed by 10% formalin at day 35 postEAE (from Figure 2B). CNS IHC was performed to assess inflammation, and myelination. Representative 40x magnification coronal images of the ventral column of thoracic spinal cord collected at day 35 postEAE. Sections collected from vehicle, IndCl, IndCl-*o*-Cl and IndCl-*o*-Me were immunostained with chemokine (C-X-C motif) ligand 1 (CXCL1; green), Glial fibrillary acidic protein (GFAP; red), and nuclear stain (DAPI; blue).

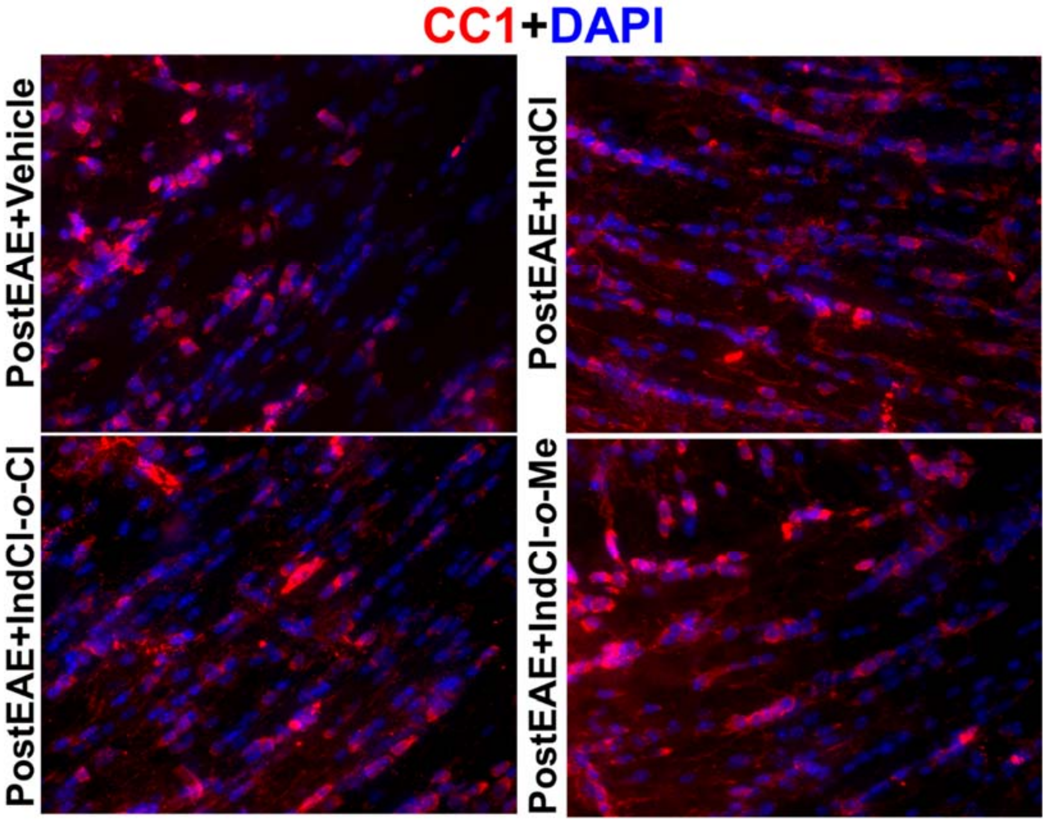

Supplementary Fig. S4. Treatment with vehicle, IndCl, IndCl-*o*-Cl, IndCl-*o*-Me was started on peak EAE disease on day 17 and continued until day 35. Mice were sacrificed and perfused with saline followed by 10% formalin at day 35 postEAE (from Figure 2B). CNS IHC was performed to assess inflammation, and myelination. Representative 40x magnification images of mature OLs in the corpus callosum of day 35 postEAE mice treated with vehicle, IndCl, IndCl-*o*-Cl and IndCl-*o*-Me. Tissues were immunostained with adenomatous polyposis coli (CC1; red) and nuclear DAPI stain (blue).

C. Detailed Materials and Methods

Primary OPC Cultures

Primary OPCs, isolated from postnatal day P1 C57BL6 male and female mouse cortices as described earlier [3]. Primary OPC were cultured into 8-well chamber slides (three wells per condition, $2.5x10^5$ cells/well) for three days to attach and five days in differentiating media (consisting of DMEM-F12 with triiodothyronine- and thyroxine-containing Sato media as well as penicillin, streptomycin, insulin, N-acetyl-L-cysteine, forskolin, ciliary neurotrophic factor, neurotrophin-3, and platelet-derived growth factor receptor α [3]. A positive control (IndCl), a negative control (Vehicle consisting of the media+EtOH mixture used to dissolve IndCl), and a normal control (differentiating media alone) were used for comparison. At the end of the treatment period, cells were fixed, stained by immunocytochemistry (primary antibody polyclonal chicken myelin basic protein (MBP, Millipore AB9348), shown in green and co-stained with nuclear stain-DAPI shown in blue), and imaged with an Olympus BX61 confocal microscope (Olympus America Inc., Center Valley, PA) at 10X magnification (3 images per well). Cells were counted using the ImageJ multipoint tool, and counts were then divided by the image area (mm^2). Average cell density for each condition was then divided by the normal condition cell density. Analysis of OL differentiation consisted of counting the number of MBP+ cells and process extensions that were longer than the respective cell-body diameter and tracking the number of highly branched MBP+ cells (with three or more processes) [4]. Statistics were performed using GraphPad Prism 6 Software (La Jolla, CA). One-way ANOVA with Tukey's posthoc test for multiple comparisons was used to generate p-values, and data are presented as mean ± SEM (with $\alpha \leq 0.05$).

Primary Astrocyte Cultures and OPC/OL Astrocyte Conditioned Media Treatment

Astrocyte cultures were prepared by chemical dissociation of the cerebral cortex from p0-p4 C57BL/6 pups[5]. Cells were plated onto cell culture flasks and cultured in Dulbecco's modified Eagle medium (DMEM) containing 10% fetal bovine serum (FBS) and penicillin–streptomycin under 5% CO2 atmosphere at 37 °C. Cultures were enriched for astrocytes after five days in vitro by the removal microglia and oligodendrocytes by shaking overnight at 37°C at 200 rpm in a table top shaker. Purified astrocyte cultures were then treated with 13 ng/ml IL-1β, 10 nM vehicle, IL-1β+IndCl-*o*-Cl, IL-1β+IndCl-*o*-Me or just media for 48 hours. Astrocyte conditioned media (ACM)

from the various conditions was used for primary oligodendrocyte culture treatment and ELISA. Purified oligodendrocyte cultures (40,000 cells/12 mm coverslip) were plated onto poly-L-lysine coated glass coverslips and maintained in N2B3 medium supplemented with 10 ng/ml PDGF-AA (Sigma) for 48 hours. After which, ACM from the various conditions was added to primary oligodendrocyte cultures with and without 100 nM CXCR2 antagonist, SB225002 (Tocris, Minneapolis, MN)) for 48 hours. Cells were then fixed and analyzed.

Enzyme-linked Immunosorbent Assay (ELISA)

CXCL1 concentrations (pg/ml) in astrocyte culture supernatant were measured usingenzyme-linked immunosorbent assay (ELISA) murine CXCL1 kit (PeproTech US, Rocky Hill, NJ) according to the manufacturer's instructions. Samples were incubated in a 96 well plate precoated with mouse monoclonal antibody against CXCL1 overnight at room temperature. After washing, plates were incubated with CXCL1 conjugate for two hours at RT. Following washing and addition of substrate solution, enzymatic reaction was stopped, and absorbance was read in a microplate reader (Bio-Rad) set to 405 nm with 605 nm wavelength corrections.

Experimental Autoimmune Encephalomyelitis (EAE)

Active EAE was induced in eight-week-old female C57BL/6 and Thy1-YFP mice as previously described [6]. Briefly, mice received two subcutaneous (s.c) injections, each consisting of MOG_{35-55} peptide (Mimotopes, Clayton, Victoria, Australia) emulsified with *M. butyricum*-containing complete Freund's adjuvant (BD Difco, Franklin Lakes, NJ) supplemented with *M. tuberculosis* (BD Difco), followed by two intraperitoneal injections of *Bordetella pertussis* toxin (List Biological Laboratories, Campbell, CA). Mice were monitored daily in accordance with standard EAE clinical disease scoring scale modified from Pettinelli and McFarlin[6,7]. Animals were maintained in accordance with guidelines set by the National Institute of Health and as mandated by the University of California Riverside Office of Research Integrity and the Institutional Animal Care and Use Committee (IACUC) in compliance with the American Association for Laboratory Animal Science (AALAS).

Drug Treatment

Normal and 6 treatment groups comprised of n=10 animals/group. Prophylactic 17β-estradiol (E2) (Sigma-Aldrich; St. Louis, MO), therapeutic E2, therapeutic chloroindazole (IndCl)[8], chloroindazole-*o*-chloro (IndCl-*o*-Cl) and chloroindazole-*o*-methyl (IndCl-*o*-Me) were dissolved in 10% ethanol and 90% Miglyol 812N (vehicle) (Cremer; Sasol, Germany). Positive control groups received a 0.1 ml subcutaneous (s.c.) injection at 0.05 mg/kg/day E2 at EAE day 0 (preEAE). Therapeutic treatment (s.c) with vehicle and various ERβ ligands at 5 mg/kg/day was initiated at EAE postinduction day 8 (postEAE; onset of clinical symptoms) and continued until day 30. Another subset of EAE mice were treated at day 17 postEAE and continued until day 35. Animals were euthanized according to the 2013 AVMA Guidelines on Euthanasia and after induction of disease were sacrificed on either on day 20-21 for flow cytometry, and luminex analysis with immunohistochemistry performed on day 21 and day 35 postEAE, and day 30 for electrophysiology.

Spleen Isolation and Cytokine Analysis

On day 20-21 after induction of EAE, spleens were harvested prior to transcardial perfusion. Splenocytes were stimulated with the autoantigen MOG_{35-55} peptide at 25 μg/ml. Supernatants were collected after 48 hours, and the levels of anti-inflammatory cytokines: IL-10, IL-13, IL-4 and IL-5; pro-inflammatory cytokines IFNγ, IL-17, IL-1β TNFα, IL-6 and IL-2; and chemokines: CXCL1, CXCL10 were determined by Cytokine Mouse Magnetic Panel for Luminex (Thermo Fisher Scientific; Waltham, MA) and run on the xMAP MAGPIX 100TM instrument (Luminex Corporation, Austin, Tx) according to manufacturer's instructions.

Rotarod behavioral assay

Motor behavior was tested up to two times per week for each mouse using a rotarod apparatus (Med Associates, Inc., St. Albans, VT). Briefly, animals were placed on a rotating horizontal cylinder for a maximum of 200 seconds. The amount of time the mouse remained walking on the cylinder without falling was recorded. Each mouse was tested on a speed of 3-30 rpm and given three trials for any given day. The three trials were averaged to report a single value for an individual mouse, and averages were then calculated for all animals within a given treatment group [9]. The first two trial days prior to immunization served as practice trials.

Histological Preparation of Tissues

Mice were deeply anesthetized by isoflurane (Piramal Healthcare) inhalation and perfused transcardially with 1x PBS followed by 10% formalin (Thermo Fisher Scientific) to fix tissues. Brains and spinal cords were dissected and post-fixed in 10% formalin (Thermo Fisher Scientific) for 24 hours, then cryoprotected in 30% sucrose (EMD Millipore, Darmstadt, Germany) for 48 hours and embedded in gelatin for sectioning. Embedded brains and spinal cords were then cut into 40-µm coronal sections using an HM525 NX cryostat (Thermo Fisher Scientific). Sections were collected serially and stored in PBS with 1% sodium azide at 4°C until staining by immunohistochemistry, following a previously described protocol[9,10].

Immunohistochemistry

Before histological staining, 40-µm free-floating sections were thoroughly washed with PBS to remove residual sodium azide [10]. Sections were permeabilized with 0.3% Triton X-100 in 1x PBS and 15% normal goat serum (NGS). Myelination, gliosis and immune markers were visualized by the following primary antibodies at a concentration of 1:500 unless otherwise noted: chicken anti-myelin basic protein (MBP; polyclonal, EMD Millipore, Darmstadt, Germany), chicken anti-glial fibrillary acidic protein (GFAP; EMD Millipore, Darmstadt), rat anti-cluster of differentiation 45 (CD45; clone 30-F11, BD Biosciences, San Diego, CA), mouse anti-ionized calcium-binding adapter molecule 1/ allograft inflammatory factor-1 (Iba1/AIF1; clone 20A12.1, EMD Millipore, Darmstadt, Germany), goat anti-CXCL1 (R&D systems; Minneapolis, MN) at 1:250 and mouse anti-adenomatous polyposis coli (CC-1; clone CC-1, Genetex, Irvine, CA). Secondary staining was performed using polyclonal fluorophore-conjugated antibodies from ThermoFisher Scientific at a concentration of 1:500 unless otherwise specified: goat anti-chicken Alexa Fluor® 555 (AF555), goat anti-rabbit Alexa Fluor®647 (AF647), donkey anti-chicken IgY Cy3 (EMD Millipore), goat anti-rat IgG AF647, goat anti-rabbit IgG Cy3 (EMD Millipore), goat anti-mouse IgG2b AF647 and rabbit anti-goat AF647. Nuclei were counter stained with 4′,6-Diamidino-2-phenylindole (DAPI, 2 ng/ml; Molecular Probes) for 10 minutes after incubation with secondary antibodies, and sections were mounted on glass slides, allowed to dry, and coverslipped with Fluoromount G mounting medium (Thermo Fisher Scientific) for imaging.

Quantification and Microscopy

Thoracic spinal cord dorsal and ventral column sections, as well as CC were imaged using an Olympus BX61 confocal microscope (Olympus America Inc., Center Valley, PA) using a 10x and 40x objective. Z-stack projections were compiled using SlideBook 6 software (Intelligent Imaging Innovations, Inc., Denver, CO). Immunostaining was quantified using unbiased stereology[10]. All images (RGB) were converted to grayscale, split, and separated by color channel using imageJ version 2.2.0-rc-46/1.50g (NIH). To avoid experimenter bias, auto-adjustment of brightness and contrast, as well as threshold of staining signal, was carried out by ImageJ. MBP^+, $GFAP^+$, $CD45^+$, and $CXCL1^+$, staining intensity was measured as percent area of positive immunoreactivity within the region of interest and intensity of signal determined by ImageJ. $CC1^+$ cell numbers were automatically counted within a region of interest using ImageJ.

Electrophysiology

To assess functional conductivity across the corpus callosum (CC), electrophysiological recordings of compound action potentials (CAPs) were measured as previously described[10,11]. Coronal brain slices corresponded approximately to plates 29–48 in the atlas of Franklin and Paxinos (2004) were prepared from adult (3 to 4 month) old C57BL/6 female mice[12]. Briefly, mice were deeply anesthetized under isoflurane and decapitated. The brain was removed and submerged in partially frozen "slushy" solution of slicing buffer containing (in mM): 87 NaCl, 75 sucrose 2.5 KCl, 0.5 $CaCl_2$, 7 $MgCl_2$, 1.25 NaH_2PO_4, 25 $NaHCO_3$, 10 glucose, 1.3 ascorbic acid, 0.1 kynurenic acid, 2.0 pyruvate, and 3.5 MOPS, bubbled with 5% CO_2 + 95% O_2[13]. Coronal slices (350 µm) were prepared using a Leica VT 1000S Vibratome (Bannockburn, IL) and subsequently incubated for 45 minutes at 35 °C in slicing buffer. Following incubation, slices were allowed to cool to room temperature for 15 minutes then transferred to artificial cerebrospinal fluid (ACSF) containing (in mM): 125 NaCl, 2.5 KCl, 2.5 $CaCl_2$, 1.3 $MgCl_2$, 1.25 NaH_2PO_4, 26.0 $NaHCO_3$, and 15 glucose, oxygenated with 5% CO_2 + 95% O_2. Slices were equilibrated in the standard ACSF for a minimum of 15-20 minutes prior to electrophysiological recordings. During electrophysiological recordings, slices were continuously perfused with oxygenated ACSF maintained at a flow rate of 1 mL/min. For recording CAPs, an Axon Digidata 1550 was used with a Multiclamp 700B Amplifier and PClamp 10.4 Software (Molecular Devices, Sunnyvale, CA). Continuous recordings for CC conduction experiments were low-pass filtered at 10 kHz and digitized at 200 kHz. All experiments

were conducted at room temperature (24-26 °C). To stimulate the CC fiber tract, a concentric bipolar stimulating electrode (FHC Neural microTargeting Worldwide, Bowdoin, ME, USA) was placed approximately 1 mm away across from a recoding electrode (glass micropipette filled with ACSF) with a resistance of 1-3 MΩ. To elicit CAPs, an episodic stimulation protocol was created consisting of 8 consecutive sweeps, each 12 ms long, with a 5-sec delay between sweeps and an immediate stimulus (0.01 ms duration) after the start of each sweep [14]. Stimulus intensity was adjusted manually using an ISO-Flex stimulator (A.M.P.I). Standardized input-output plots were generated in current clamp mode for each slice by averaging at least 4 consecutive sweeps together to reduce the signal-to-noise ratio. Brain slices that exhibited near zero voltage even when stimulated with the maximal current were not included in the analysis.

Statistical Analysis

All statistics were performed using Prism 6 software (GraphPad Software, La Jolla, CA). Differences in EAE clinical scores were determined by two-way unbalanced ANOVA with Dunnett's multiple comparisons test[6]. Luminex data and immunohistochemistry data were analyzed by ordinary one-way ANOVA with Dunnett's multiple comparisons test either if data satisfied assumptions of normal distribution (D'Agostino & Pearson omnibus normality test) and equal variances among all groups or Kruskal Wallis with Dunn's multiple comparisons test. CAP recording analysis was carried out per previously published work[9,11] using Clampfit 10.4 software (Molecular Devices, Sunnyvale, CA), OriginPro 2016 64Bit (OriginLab Corporation) and GraphPad Prism 6 (GraphPad Software). The averaged mean amplitude was compared using one-way ANOVA with post hoc tests using Tukey's multiple comparison test. All data are presented as mean ± SEM for two independent experiments. Differences were considered significant at * $p \leq 0.05$, ** $p \leq 0.01$, and *** $p \leq 0.001$, **** $p \leq 0.0001$.

D. References

1 Carlson, K. E., Choi, I., Gee, A., Katzenellenbogen, B. S. & Katzenellenbogen, J. A. Altered ligand binding properties and enhanced stability of a constitutively active estrogen receptor: evidence that an open pocket conformation is required for ligand interaction. *Biochemistry* **36**, 14897-14905, doi:10.1021/bi971746l bi971746l [pii] (1997).

2 Katzenellenbogen, J. A., Johnson, H. J., Jr. & Carlson, K. E. Studies on the uterine, cytoplasmic estrogen binding protein. Thermal stability and ligand dissociation rate. An assay of empty and filled sites by exchange. *Biochemistry* **12**, 4092-4099 (1973).

3 Tiwari-Woodruff, S. K. *et al.* OSP/claudin-11 forms a complex with a novel member of the tetraspanin super family and beta1 integrin and regulates proliferation and migration of oligodendrocytes. *The Journal of cell biology* **153**, 295-305 (2001).

4 Monnerie, H. *et al.* Reduced sterol regulatory element-binding protein (SREBP) processing through site-1 protease (S1P) inhibition alters oligodendrocyte differentiation in vitro. *J Neurochem* **140**, 53-67, doi:10.1111/jnc.13721 (2017).

5 Schildge, S., Bohrer, C., Beck, K. & Schachtrup, C. Isolation and culture of mouse cortical astrocytes. *J Vis Exp*, doi:10.3791/50079 (2013).

6 Hasselmann, J. P. C., Karim, H., Khalaj, A. J., Ghosh, S. & Tiwari-Woodruff, S. K. Consistent induction of chronic experimental autoimmune encephalomyelitis in C57BL/6 mice for the longitudinal study of pathology and repair. *J Neurosci Methods* **284**, 71-84, doi:10.1016/j.jneumeth.2017.04.003 (2017).

7 Pettinelli, C. B. & McFarlin, D. E. Adoptive transfer of experimental allergic encephalomyelitis in SJL/J mice after in vitro activation of lymph node cells by myelin basic protein: requirement for Lyt 1+ 2- T lymphocytes. *Journal of immunology* **127**, 1420-1423 (1981).

8 De Angelis, M., Stossi, F., Carlson, K. A., Katzenellenbogen, B. S. & Katzenellenbogen, J. A. Indazole Estrogens: Highly Selective Ligands for the Estrogen Receptor β. *Journal of Medicinal Chemistry* **48**, 1132-1144, doi:10.1021/jm049223g (2005).

9 Moore, S. M. *et al.* Multiple functional therapeutic effects of the estrogen receptor beta agonist indazole-Cl in a mouse model of multiple sclerosis. *Proc Natl Acad Sci U S A* **111**, 18061-18066, doi:10.1073/pnas.1411294111 (2014).

10 Crawford, D. K. *et al.* Oestrogen receptor beta ligand: a novel treatment to enhance endogenous functional remyelination. *Brain : a journal of neurology* **133**, 2999-3016, doi:10.1093/brain/awq237 (2010).

11 Crawford, D. K., Mangiardi, M., Xia, X., Lopez-Valdes, H. E. & Tiwari-Woodruff, S. K. Functional recovery of callosal axons following demyelination: a critical window. *Neuroscience* **164**, 1407-1421, doi:10.1016/j.neuroscience.2009.09.069 (2009).

12 Paxinos, G. & Franklin, K. B. J. *The Mouse Brain in Stereotaxic Coordinates*. (Elsevier Academic Press, 2004).

13 Lauderdale, K. *et al.* Osmotic Edema Rapidly Increases Neuronal Excitability Through Activation of NMDA Receptor-Dependent Slow Inward Currents in Juvenile and Adult Hippocampus. *ASN Neuro* **7** (2015).

14 Crawford, D. K., Mangiardi, M. & Tiwari-Woodruff, S. K. Assaying the functional effects of demyelination and remyelination: revisiting field potential recordings. *J Neurosci Meth* **182**, 25-33, doi:10.1016/j.jneumeth.2009.05.013 (2009).
